# Spillovers and contagion between BRIC and G7 markets: New evidence from time-frequency analysis

**DOI:** 10.1371/journal.pone.0271088

**Published:** 2022-07-27

**Authors:** Samuel Kwaku Agyei, Peterson Owusu Junior, Ahmed Bossman, Emmanuel Asafo-Adjei, Oliver Asiamah, Anokye Mohammed Adam

**Affiliations:** 1 Department of Finance, School of Business, University of Cape Coast, Cape Coast, Ghana; 2 Laboratoire d’Analyse et de Prospective Economiques, Universite de Limoges, Limoges, France; Newcastle University, UNITED KINGDOM

## Abstract

We examine the time-frequency spillovers, contagion, and pairwise interrelations between the BRIC index and its constituents, and between BRIC and G7 economies. The extent of interdependencies between market blocs and their constituents needs to be ascertained in the time-frequency domain for efficient asset allocation and portfolio management. Accordingly, the Baruník and Křehlík spillover index is employed with daily data between 11^th^ December 2015 and 28^th^ May 2021. We find the overall and net spillovers between BRIC and G7 to be significant in the short-term, with France, Germany, and the UK transmitting the greatest shocks to BRIC markets. We find no significant evidence of any sporadic volatilities for the studied markets in the COVID-19 period across all frequencies. However, we reveal contagious spillovers between the BRIC and G7 economies across all time scales in 2017 and 2019, which respectively reflect the persistent effect of Brexit and the US-China trade tension. Our findings divulge that in the short-term (mid-to-long-term), France and the UK (Canada and the US), are the sources of contagion between the BRIC and G7 markets. From the net-pairwise spillovers, we report high connectedness between the BRIC index and its members. BRIC countries are found to be transmitters of net-pairwise spillovers to the G7 markets excluding Japan. We recommend portfolio diversification using BRIC and G7 stocks in the intermediate-to-long-term horizon, where spillovers are less concentrated. Additionally, since individual markets are impacted by their unique shocks, investors should pay close attention to these shocks when distributing assets. In the interim, policy-makers and governments across the globe should ensure effective liberalisation of their economies to encourage international trade flows to boost portfolio diversification.

## 1. Introduction

Since O’neill’s [1] coining of the term ‘BRIC,’ the world’s main middle-income and developing nations of Brazil, Russia, India, and China have pursued increasingly divergent pathways to economic growth [2]. After the 2007/08 Global Financial Crisis (GFC), the concept of the BRIC as synchronised engines of global development disappeared as the BRIC established relatively diverse investment markets such as market size and scope, and institutional maturity [3]. Consequently, the portfolio risk diversification features of BRIC equity markets transformed [4].

Notwithstanding, BRIC economies continue to affect the global financial stability setting [5], due in part to the growth of risks of bi-directional spillovers between developed and developing markets [6]. Thus, as the principles of the portfolio selection theory [7] detail, investors from developed markets–which are known to be more integrated–almost always seek assets from developing and emerging economies–which are relatively less integrated–to maximise (minimise) portfolio returns (risk); this has seen the spillovers between these markets increasing. Economists and experts alike advocate that these spillovers are highly heterogeneous [8–11]. On the one hand, China could lead international markets and has greater contagion pathways into international financial markets. Conversely, Russia appears to have decoupled greatly from worldwide financial flows volatility and patterns, particularly after the sharp decline in US–Russia geopolitical ties began in 2011. While these insights are prevalent in media and political science research, they have yet to be thoroughly investigated in financial literature on volatility and risk spillovers [10].

The focus of this research is to show how the composite BRIC market–owing to market integration–and particular BRIC markets are linked or interrelated with developed economies markets regarding risk spillover pathways, as well as how these interrelations change across investment horizons and during episodes of overall market turmoil and recovery.

Against this backdrop, we propose some key preliminary contributions to the body of knowledge on the dynamic study of BRIC-to-advanced-economy contagion routes which is expanded in subsequent paragraphs. In our study of contagion, we introduce the composite BRIC index and analyse it against its constituents and developed markets. Also, we extend our analysis to the COVID-19 era, which given the channels of effect, presents somewhat a unique financial crisis globally [12,13]. Methodologically, we employ a unique and novel econometric approach, the Baruník and Křehlík [14] (hereafter, BK-18) spillover index. No present study integrates these two aspects of analysis while using the BK-18 methodology.

The study–at this time and the focus on the BRIC–is essential for notable reasons. The BRICS (including South Africa) equity markets have continuously provided strong average returns and evolving correlations with developed markets; a feature that is significant for risk mitigation and allows for more globally diversified portfolios to be built [15]. According to Barry, Peavy, and Rodriguez [16], certain developing and emerging economies will become developed economies in the future, which now pertains to the BRIC(S) countries [4]. Corollary to these characteristics, emerging markets have appeared as a significant asset class, and their inclusion in international and specialised portfolios is becoming increasingly crucial, as they provide substantial diversification benefits for investors in advanced economies.

Additionally, globalisation and financial liberalisation have also increased the liquidity of developing markets, expanded the breadth and depth of their markets, and bolstered investor confidence, particularly for minority stockholders. It is in this direction that Buchanan, English and Gordon [17] emphasise the relevance of adding developing markets (such as the BRIC) to advanced market portfolios since it allows investors to attain better risk-adjusted returns.

Notwithstanding, high volatility has been a feature of financial markets, particularly during instances of structural disruption, such as the COVID-19 pandemic, the Brexit effect (we define the Brexit effect as the substantial losses borne by global investors on 24 June 2016 following the referendum that confirmed Britain’s exit (Brexit) from the European Union [64]), the GFC, and the 2011/12 Eurozone debt crisis (EDC). During bullish and bearish markets, these markets also display nonlinear behaviour in reaction to direct and inverse shocks, resulting in portfolio rebalancing as a result of shifting co-movements [4,5,18–20]. Appreciating the volatility behaviour of equity markets amid significant events and crises, especially the time-varying spillover connectedness between the equity markets of most influential emerging economies, such as the BRIC, and major advanced economies, is a principal challenge for multinational investors and policymakers [21]. The aforementioned validations reignite the essence of this study at this time, focusing not only on equities from developing markets but also incorporating a group of developed markets.

This study examines the matter of spillovers and contagion effects between the BRIC index and its members, and the G7 markets while taking into account the impacts of the adaptability [22] and heterogeneity [23–25] of market participants, non-linearity and asymmetric qualities (of spillovers) that have not been properly considered in prior studies. As a result, the primary goal of this research is to look at the time-varying spillovers between those four fast-growing nations, their market bloc index, and the group of seven (G7) major developed stock markets. The origins and degree of contagion are then assessed, which might give significant insights into the financial implications of these equity markets’ dynamic linkages for portfolio risk management and the relevance of effective international trade flows liberalisation.

It is instructive to note that although the markets of BRIC and G7 are distinct in diverse phases, the recent growth in emerging markets and the new alliances formed between developing economies explicates their potential of becoming future developed markets [16] and this is not far from BRIC markets [4]. Notwithstanding, increasing growth and alliances, which result in the liberalisation of economies, increase the chance of economic and financial market integration [26], which could easily wipe off diversification advantages associated with assets from emerging markets. Invariably, capital flows and direct foreign investment in emerging markets may be impacted and could further translate into worsened stock market performance.

Indicatively, the ratio of BRIC GDP to global GDP increased from 0.12 in 2009 to 0.236 in 2019 [27]. In the future, BRIC’s effect on the global economy may surpass that of the G7. The dynamic interaction between the financial markets of the BRIC countries and the G7 countries has gotten a lot of attention recently [5,27–30]. International investors are particularly interested in the idea of BRIC replacing the G7 as a major source of international risks since this might lead to extended market integration which may alter investment, speculation, and risk diversification options [27]. Assessments of cross-market linkages covering past financial crises may have minimal effects on portfolio management owing to the distinct nature of recent systemic crises [13,23,24]. As a result, focusing on the time-varying dynamics of cross-market linkages in periods such as the EDC, Brexit, the US-China trade tension, and the financial market meltdown in the COVID-19 era is essential to understand emerging trends in financial markets. From this backdrop, we maintain that analysing the time-varying spillover, directional, and pairwise linkages between and among the BRIC market and the group of seven major developed stock markets (i.e., G7) is timely.

The study makes at least four significant contributions to the body of knowledge. First, we analyse spillovers and contagion between the BRIC constituents’ equity indices and the BRIC composite index together with G7 indices across time and frequencies. This helps disclose the level of heterogeneity among them by determining the spillover connectedness of each BRIC constituent to the BRIC composite index and G7 indices. Second, within distinct frequency bands, the dynamic characteristics of volatility spillover among equity markets in the composite BRIC and its constituents’ indices as well as G7 indices are examined. In practice, since systemic spillovers jeopardise the global stock market system’s stability, pinpointing the frequency-specific origin of turbulence is critical for policymakers seeking methods to track these detrimental impacts. We decompose the aggregated spillover across BRIC and G7 stock markets into frequency-specific spillovers, pinning down the dominating risk transmitters and recipients.

Third, in the frequency domain, we present net pairwise spillovers, which give novel ideas for portfolio managers looking to allocate equities across the BRICS and G7 markets. Diversification opportunities differ at various frequencies, as Tiwari, Cunado, Gupta, and Wohar [31] revealed. Fourth, we incorporate the time-varying market players’ emotions, expectations, and risk preferences in our analysis by looking at how the complex connections–both aggregate and pairwise–between the various BRIC constituents and G7 stock markets have evolved through time and frequencies. These assessments are critical for optimal investment decisions by all classes of investors with varying risk appetites.

In terms of the econometric approach, we use the BK-18 methodology, which builds upon the Diebold and Yilmaz [32] (hereafter, DY-12) approach. The DY-12 spillover index rather assumes a time-invariant connectedness between assets, suggesting that investors respond to spillovers similarly and that their response is unaffected by investment horizons. This is contrary to the heterogeneous markets hypothesis (HMH) [23–25,33] and adaptive market hypothesis (AMH) of Lo [22]. To get around this restriction, we employ the BK-18 spillover index, which is based on heterogeneous shock frequency responses. The BK-18 index offers valuable information on the intensities and directions of spillovers in the time-frequency domain. This approach is essential for determining the source and magnitude of contagions, and the market receiver(s) and transmitter(s) of shocks across different investment horizons, represented by frequency bands. The suggested methodology, by way of revealing the dynamic, directional, and net pairwise spillovers, helps to answer questions relating to the source(s) and magnitude of contagious spillovers whilst revealing potential diversifiers, hedgers, and safe-havens across distinct market periods and economic or investment timescales (i.e., short-, medium-, and long-term). Additionally, the method is capable of producing results that help in assessing the relative relevance of transitory spillovers.

By isolating the frequency domain spillover effects from the aggregate risk spillover effects, this decomposition provides essential benefits to investors Tiwari et al. [31]. Fund managers and investors may optimise their financing allocation and hedge their position against severe falling prices by distinguishing the time horizons. The notion that market players work on distinct investment horizons is primarily motivated by the development of investor risk appetites.

The results divulge that spillovers between emerging and developed markets are concentrated at high frequencies (short-term). No significant contagion is spotted COVID-19 pandemic era, but one in 2017 and 2019 owing to Brexit and the US-China trade tension, respectively. We find diversification opportunities between BRIC and G7 markets in the intermediate- to long-term horizon, where the spillovers are mild. Notably, the constituents of BRIC significantly integrate toward the BRIC index. We find that BRIC markets stand the chance to transmit spillovers to G7 markets save Japan.

The remainder of this paper is structured under Sections 2–6. In Section 2, we summarise existing works related to our study; we detail the methodology in Section 3; data and preliminary results are presented in Section 4; we report the study’s findings in Section 5; practical implications are emphasised in Section 6; we conclude and present recommendations in Section 7.

## 2. Literature review

### 2.1. Theoretical background

Heterogeneous and time-based investor behaviour is reflected in market pricing since the market does not function in solitude. Theories that support this occurrence are Lo’s [22] AMH and the HMH recently propagated by Adam, Gyamfi, Kyei, Moyo, and Gill [25], Bossman [23], Bossman et al. [24], and Owusu Junior, Frimpong et al. [34]. By analysing historical and present events, the HMH hypothesises that distinct economic actors make investment decisions over different time horizons depending on their risk and return preferences. To account for time horizon, we could define time as intrinsic time, which relates to short-, medium-, and long-term horizons [18–20]. During market stress, the adaptive and heterogenous behaviour of the market encourages investors to move to or incorporate diverse asset classes in their portfolios for hedging and risk diversification.

Prior episodes of financial crises have caused a spate of research to revisit the fundamental notion of portfolio diversification in pursuit of competitive returns and risk levels. Several studies support the idea that investors are continually seeking contrasting risks and returns, and that this search intensifies in stressful market conditions [8,9,35–38]. Furthermore, although information travels across markets as a consequence of investor queries, information flows become more intense during times of market stress. This situation exemplifies Owusu Junior, Frimpong et al.’s [34] competitive market hypothesis (CMH), which states that the intensity of information flows and spillover between markets of the same and different asset classes is exacerbated in part by rational, albeit rather irrational, investors’ never-ending search for competing rewards and risks to meet portfolio goals.

As already indicated, assets from emerging economies tend to be chiefly considered in multinational portfolios due in part to the growth of risks of bi-directional spillovers between advanced and emerging markets [6]. The returns offered by assets from emerging markets are more consistent relative to those from advanced markets [4]. Thus, emerging markets have appeared as a significant asset class, and their inclusion in universal and dedicated portfolios is becoming increasingly vital, as they provide considerable diversification opportunities for all classes of investors. It is worth noting, however, that greater portfolio diversity requires in-depth knowledge of the co-movements, interdependencies, and spillovers among asset classes. The majority of studies are limited to either developed markets or the BRIC markets only.

### 2.2. Empirical review

As noted earlier, scholarly attention on the relationship between emerging and developed markets in recent times is driven by the high propensity for the top-emerging markets to become developed markets. This has seen studies comparing the top developed and emerging markets. Notable works include [10,27,28,39–41]. In a static spillover paradigm, Zhang et al. [27] analyse the connectedness of G7 and BRIC economies. However, the operability of markets and investor behaviour are shown to follow dynamic patterns and, hence, evidence from static connectedness measures may be insufficient for time-based investors operating across the short-, medium-, and long-term periods [5,11,23–25,34]. Analysis of safe-haven assets [39], risk-aversion comovements [40], spillovers dynamics between developed and emerging economies, and commodity futures [28] have all been studied with much emphasis on G7 markets and to some extent, BRIC economies. Thus, probing into the total, directional, and paired connectedness between developed and emerging economies in a time-frequency domain paradigm is essential for portfolio and risk management amid systemic risk periods [23,24,42–50].

The extant literature on BRIC markets can be classified into at least four notable strands. The first strand assesses the sectoral or market-wide connectedness of BRIC economies, leading Ahmad, Mishra, and Daly [51] to explore, at the sectoral level, the dynamic reliance structure between BRIC and international markets (Europe, the US, and World) via return and volatility spillovers. They show strong evidence of variability across sample sectors within BRIC and worldwide indices using directional spillover and dynamic conditional correlation models. Ahmad et al. [51] divulge that among the BRIC, the Chinese and Indian sectoral indices offer greater risk management prospects. As a result of the diversified reliance structure, the authors infer that BRIC is a unique asset class in terms of strategic portfolio management. Panda and Thiripalraju [2] examine spillovers among the BRICS markets from 26/06/2002 to 31/07/2014 under the exponential GARCH framework. The authors discover bidirectional and unidirectional spillover between the studied markets. They divulge that diversification does not provide any economic value from the stock markets of Brazil, Russia, and India, to that of China. This conclusion may suggest some form of integration among BRICS markets.

The second strand focuses on BRIC markets and the US market or developed markets. Given that BRIC economies may be integrated, sliming diversification prospects between them, the introduction of international assets may be deemed essential for optimal portfolio management. This influences other studies which consider the US and/or other developed markets in examining their diversification prospects with the BRIC economies. Gurdgiev and O’Riordan [10] employ the wavelet methodology to assess contagion between the US and BRIC markets with data from 2000 to 2016. Their findings corroborate those in sparse literature that describe the time-varying and uneven integration of BRIC markets with advanced economies. Panda, Vasudevan, and Panda [29] examine the time-varying co-movement between BRICS and developed equity markets using the DY-12 spillover index. With data from 02/08/2002 to 28/12/2017, the authors find a doubling of volatility spillovers during the GFC period.

The role of the volatility index (VIX) in the BRIC markets’ connectedness dominates the third strand. Sarwar [52] examines the time-inconsistent connections between the CBOE VIX and stock returns for the BRIC economies where an inverse relationship between BRIC stocks and the VIX was revealed. More recently, Owusu Junior, Adam et al. [5] incorporate the VIX in evaluating the time-varying co-movements between BRIC markets using the wavelet methodology and reveal findings that corroborate the negative role of the VIX in the co-movements of BRIC markets. Their study commenced the prevailing (fourth) strand of the BRIC literature, focusing on the composite BRIC index and its constituents. Owusu Junior, Adam et al. [5] considered the time-frequency co-movements between the composite BRIC index and its members, which was novel to the existing literature vis-à-vis the BRIC market bloc.

### 2.3. Motivation

We note from the reviewed literature that the individual BRIC markets are mostly compared with only the US on the premise that a one-way impact is transmitted from the US economy to other nations [2,21] and “when America sneezes, the whole world catches a cold” [53]. Also, the studies on spillovers have failed to extend to the COVID-19 pandemic era, which according to Quinsee [13], drives a financial crisis caused by exogenous shocks rather than endogenous shocks that caused other crises like the 2007/08 GFC. Recent events, like Brexit, the US-China trade tension, and the financial hardships driven by the COVID-19 pandemic necessitate the inclusion of developed economies in the analysis of volatility spillovers between middle-income and developing economies. Thus, following the emerging strand of research concerning the BRIC economies pioneered by Owusu Junior, Adam et al. [5], we analyse the individual stocks of BRIC, the composite BRIC index, and G7 economies to assess the relative intensity of spillovers among them, and the source(s) of contagion.

Regarding the methods, the extant literature has been predominant with dynamic equicorrelation-fractionally integrated exponential GARCH [4], exponential GARCH [2], VAR-BEKK [54], dynamic equicorrelation GARCH [55], wavelets [10], BEKK-GARCH [56], DY-12 spillover index [21,29,51]; directed acyclic graph-SVAR [27]. These approaches, e.g., the GARCH techniques, VAR, and wavelets, could at best only determine spillovers and contagion, but cannot assess their magnitude and source(s). On the other hand, the DY-12 spillover index does not reflect the adaptive, dynamic, and complex nature of investor behaviour–as expounded by the AMH and HMH–because it assumes a static connection between variables over time.

Corollary to the aforementioned limitations, we employ a novel approach–the BK-18 spillover index, which caters for non-linearity, asymmetries, and complexity in investor behaviour–to model and examine the overall, pairwise, and net spillovers and contagion between the composite BRIC index, its constituents, and the G7 indices. This is non-existent in the extant literature. We cover a relatively long period that captures the periods of the EDC, Brexit, the US-China trade tension, and more importantly the COVID-19 pandemic. By its application, the method enables the study to provide answers to questions relating to the source(s) and magnitude of contagion and at the same time reveal potential diversifiers and hedgers, and safe-haven during average and stressed trading conditions, respectively across distinct investment horizons (i.e., short-, medium-, and long-term). Furthermore, findings from the method would help assess the relative relevance of transitory spillovers against their permanent counterparts.

## 3. Methods

The Baruník and Křehlík [14] spillover index is employed to examine the dynamic connectedness, spillovers, and contagion between the BRIC and G7 equities markets to reveal the time-frequency dynamics of emerging and developed equities. The BK-18 spillover index is detailed as follows:

Baruník and Křehlík [14] employ generalised forecast error variance decompositions (GFEVDs) to quantify co-movements, as inspired by Diebold and Yilmaz [32]. Data is decomposed using the matrix of a vector autoregressive (VAR) model with local covariance stationarity. We represent a *K*-variate procedure, *Y*_*t*_ = (*y*_1,*t*_,…,*y*_*K*,*t*_)′ given *t* = 1,…,*T* and a *VAR*(*p*) which may be expressed as

$$Y_{t}=\sum_{i=1}^{p}{\phi_{i}Y}_{t-i}+\epsilon_{t},$$ (1)

where coefficient matrices and white noise with (prospective non-diagonal) covariance matrix Π are denoted as *ϕ*_*i*_ and *ϵ*_*i*_. A regression is carried out between each variable in the system (1) and its p lags, and the p lags of the remaining variables. Accordingly, *ϕ* holds wide-ranging information on the relationships between all variables. The suitability of working with a (*K*×*K*) matrix (***I***_*K*_−∅_1_*L*−⋯−∅_*p*_*L*^*p*^) with identity ***I***_*K*_ must be noted. The VAR system is circumscribed by a moving average *MA*(∞) when the roots of the representative equation |*θ*(*z*)| lie outside of the unit circle

$$Y_{t}=\psi (L)\epsilon_{t},$$ (2)

with *ψ*(*L*) depicting an infinitely lagged polynomial. The role of the *kth* variable, known as the GFEVD, to the variance of forecast error of the element *j* can be written as

$${{(\Theta }_{H})}_{j,k}=\frac{\sigma_{kk}^{-1}\sum_{h=0}^{H}{\left({\left(\psi_{h}\Pi \right)}_{j,k}\right)}^{2}}{\sum_{h=0}^{H}{\left(\psi_{h}\Pi \psi_{h^{\prime }}\right)}_{j,j}},$$ (3)

where *h* = 1,…,*H* and *σ*_*kk*_ = (Π_*kk*_). This could hold since the measure of connectedness is conditional on decomposed variance, which are the transformations of *ψ*_*h*_ and serve as the contribution of the shocks to the system. Row contributions do not sum up to 1, so, owing to completeness, a standardised matrix Θ_*H*_ is generated as

$${{(\tilde{\Theta }}_{H})}_{j,k}=\frac{{{(\Theta }_{H})}_{j,k}}{\sum_{k=1}^{N}{{(\Theta }_{H})}_{j,k}}.$$ (4)

The pairwise connectivity (4) may be aggregated for overall connectedness in a system. Following [32], this is defined as the proportion of variation in predictions provided by errors other than own error (which is the same as the ratio of the off-diagonal components’ sum to the whole matrix’s sum.) as shown in

$$C_{H}=100*\frac{\sum_{j\neq k}{{(\tilde{\Theta }}_{H})}_{j,k}}{\sum {\tilde{\Theta }}_{H}}=100*\left(1-\frac{Tr\{{\tilde{\Theta }}_{H}\}}{\sum {\tilde{\Theta }}_{H}}\right),$$ (5)

where *Tr*{.} represents the operator for tracing, and the arithmetic aggregate of all elements in the matrix is the denominator. As a result, connectedness denotes the forecast variance’s relative contribution to the system’s other variables. As a result, bi-directional connectedness may be assessed (*“to”* and/or *“from”* market *i* from all other markets *k*). The difference between *“to”* and *“from”* spillovers is also used to calculate *“net”* connectivity. Resultantly, a market with a positive net spillover acts as a “net transmitter,” while one with a negative spillover acts as a “net receiver” of shocks.

The spectral representation of connectivity is shown at this point. With a frequency response function of ${\psi (e)}^{-i\omega }=\sum_{h}e^{-i\omega h}\psi_{h}$ of coefficients that could be transformed by Fourier transforms *ψ*_*h*_ with $i=\sqrt{-1}$, a spectral density of *Y*_*t*_ at frequency, *ω*, can be defined as *MA*(∞) filtered series

$$S_{y(\omega )}=\sum_{h=-\infty }^{\infty }E(Y_{t}Y_{t-h}^{\prime })e^{-i\omega h}=\psi {(e}^{-i\omega })\Pi \psi^{\prime }{(e}^{+i\omega }),$$ (6)

where *S*_*y*(*ω*)_ is the power spectrum which details the distribution of the variance of *Y*_*t*_ over the frequency components *ω*. The causation spectrum over *ω*∈(−*π*, *π*) is defined in (7); noting that it reflects the fraction of the *ith* variable attributable to shocks in the *kth* variable at a particular frequency *ω*. Consequently,

$$({F(\omega ))}_{j,k}=\frac{\sigma_{kk}^{-1}{{|\psi {(e}^{-i\omega })\Pi }_{j,k}|}^{2}}{{\left(\psi {(e}^{-i\omega })\Pi \psi^{\prime }{(e}^{+i\omega })\right)}_{j,j}}$$ (7)

could be understood as *within-frequency* causation due to the denominator. For a natural decomposition of GFEVD to frequencies, we weigh $({F(\omega ))}_{j,k}$ by the frequency share of the variance of the *jth* variable. We define the weighting function as

$$\Gamma_{j}=\frac{{\left(\psi {(e}^{-i\omega })\Pi \psi^{\prime }{(e}^{+i\omega })\right)}_{j,j}}{\frac{1}{2\pi }\int_{-\pi }^{\pi }{\left(\psi {(e}^{-i\lambda })\Pi \psi^{\prime }{(e}^{+i\lambda })\right)}_{j,j}d\lambda }$$ (8)

summating to real-valued numbers up to 2*π* and represents the index of the *jth* variable at a particular frequency. According to Baruník and Křehlík [14], the generalised causation spectrum is the squared modulus of the weighted complex numbers, resulting in a real-valued quantity. Connectivity must be measured across periods in practical financial applications. As a result, rather than measuring connectedness at single frequencies, it is more appropriate to do so across frequency bands. We take a formal representation of frequency band, *d*, as *d* = (*a*, *b*): *a*, *b*∈(−*π*, *π*), *a*<*b*, for which we define the GFEVDs as

$$({\Theta_{d})}_{j,k}=\frac{1}{2\pi }\int_{a}^{b}{\Gamma_{j}(\omega )(F(\omega ))}_{j,k}d\omega .$$ (9)

A scaled generalised variance decomposition may be constructed in the same frequency band *d* as

$${{(\tilde{\Theta }}_{d})}_{j,k}=\frac{({\Theta_{d})}_{j,k}}{\sum_{k}{\left(\Theta_{d}\right)}_{j,k}},$$ (10)

As seen in Eqs (5), (11) and (12), the scaling factor is 100. In the practical application of the connectedness in the BK-18 framework, it is also the minimal forecast horizon *H*.

Then, the *within-frequency* and frequency connectivity across *d* are expressed in (11) and (12), respectively.


$$C_{d}^{W}=100.\left(1-\frac{Tr\{{\tilde{\Theta }}_{d}\}}{\sum {\tilde{\Theta }}_{d}}\right)$$ (11)

$$C_{d}^{F}=100.\left(\frac{\sum {\tilde{\Theta }}_{d}}{\sum {\tilde{\Theta }}_{\infty }}-\frac{Tr\{{\tilde{\Theta }}_{d}\}}{\sum {\tilde{\Theta }}_{\infty }}\right)=C_{d}^{W}.\left(\frac{\sum {\tilde{\Theta }}_{d}}{\sum {\tilde{\Theta }}_{\infty }}\right)$$ (12)

It is important to note that $C_{d}^{W}$ represents the connectedness that occurs inside a frequency band and is only weighted by the series’ power on that frequency band. $C_{d}^{F}$, on the other hand, breaks down overall connectedness into discrete pieces that add up to the original connectedness metric, as presented by Baruník and Křehlík [14]. (*π*+0.00001, *π*/4, *π*/8, *π*/32, *π*/64, 0) are the frequency bands we utilise, which is in line with Baruník and Křehlík [14], Tiwari et al. [31], Tiwari, Shahbaz, Hasim, and Elheddad [57], and Owusu Junior, Alagidede, and Tweneboah [58]. Frequency bands *d*_1_(3.14~0.79), *d*_2_(0.79~0.39), *d*_3_(0.39~0.10), *d*_4_(0.10~0.05), and *d*_5_(0.05~0.00) correspond to daily bands of 1~4 (Intraweek), 4~8 (Intraweek-to-week), 8~32 (Fortnight-to-month), 32~64 (Month-to-quarter), and 64~∞ (Quarter-and-beyond), respectively.

## 4. Data and preliminary analysis

In addition to the BRIC composite index, we employ the daily stock market indices at close for its constituents (Brazil, Russia, India, and China), and the G7 (Canada, France, Germany, Italy, Japan, the UK, and the US) markets in our analysis. The specific market indices used were: NASDAQ–BRIC, Italy, Japan, and the UK; Brazil–IBOVESPA; Russia–Moscow Exchange; India–NIFTY 500; China–Shanghai Stock Exchange; Canada–Toronto S&P/TSX; France–CAC 40; Germany–DAX. The data set spanned between 11/12/2012 and 28/05/2021, yielding 1,652 common observations. The daily stock indices were supplied by EquityRT and are expressed in USD. The log-returns of the daily stock indices were computed as *r*_*t*_ = *lnP*_*t*_−*lnP*_*t*−1_, where *r*_*t*_ defines the continuously compounded returns, *P*_*t*_ represents the price of an asset in period *t*, and *P*_*t*−1_ represents the price of an asset in the previous period *t*−1.

**Fig 1:**
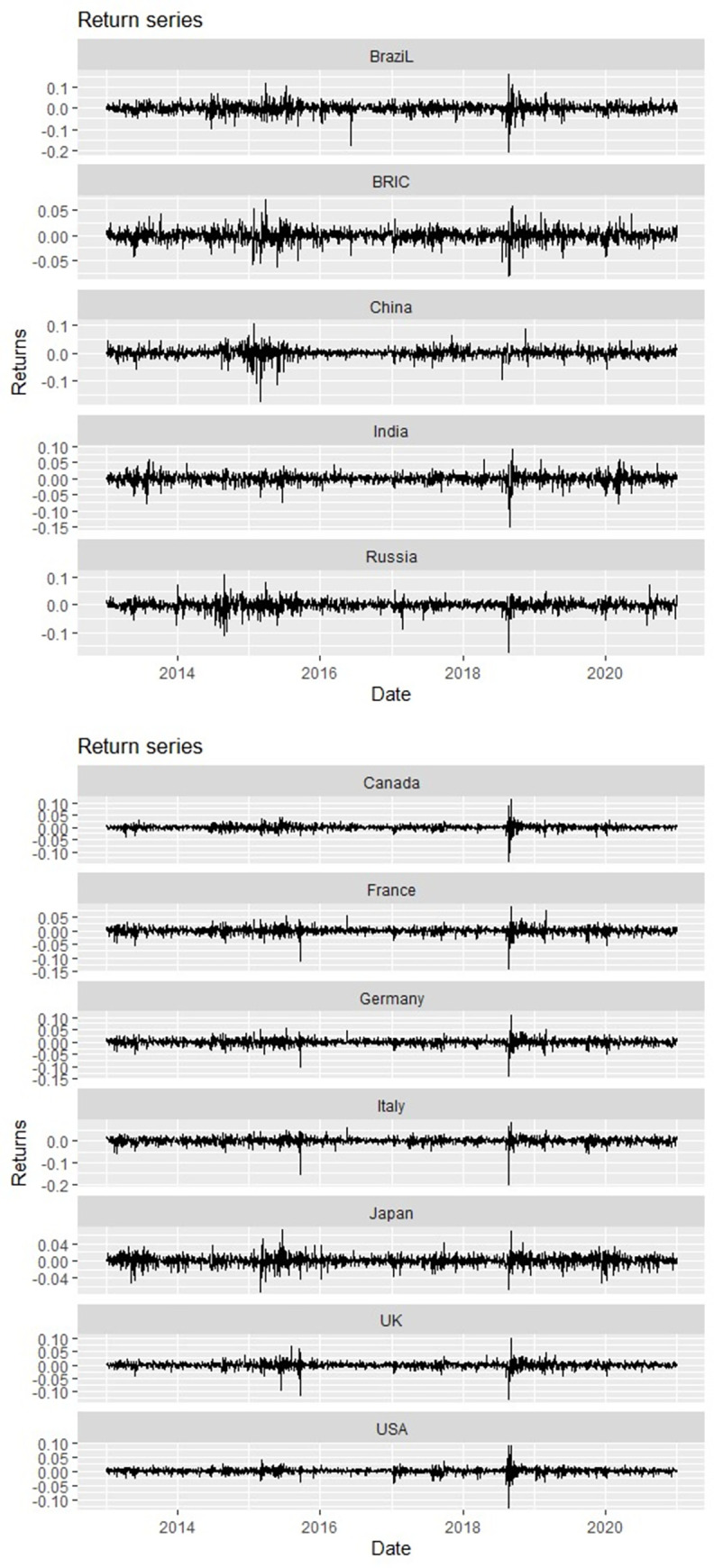
Return series plots for BRIC and G7 stock markets.

A 100-day forecast horizon (*H*) and rolling window periods are specified. The set window aggregates to a little over a quarter of a year, which is ample to accommodate for time variations. The rolling window paradigm eliminates the exogenous specifications of crisis start and end dates. Through the resultant spillover indices, we can account for significant changes in the form of spillovers throughout the sample period, as advocated by Yilmaz [59] and Owusu Junior, Alagidede et al. [14]. A trajectory of the return series is presented in Fig 1. The volatility clusters spotted in the return series plots confirm the stylised facts vis-à-vis asset returns.

We report the descriptive summary of the returns on the studied stock markets in Table 1. The results confirm skewness and excess kurtosis. The resulting statistics for skewness and kurtosis respectively depict non-normal and leptokurtic distributions across the BRIC and G7 markets.

**Table 1:** Descriptive statistics.

	Obs.	Min.	Max.	Mean	Std. Dev.	Skewness	Kurtosis	Normtest.W
**BRIC Indices**								
BRIC	1652	-0.0816	0.0723	0.0001	0.0125	-0.6692	6.2922	0.9279***
Brazil	1652	-0.2038	0.1608	-0.0003	0.0238	-0.9647	11.0980	0.9014***
Russia	1652	-0.1715	0.1094	-0.0002	0.0176	-1.0776	9.6761	0.9137***
India	1652	-0.1503	0.0897	0.0004	0.0144	-1.1087	11.8427	0.9049***
China	1652	-0.1719	0.1003	0.0003	0.0160	-1.3522	14.5403	0.8702***
**G7 Indices**								
Canada	1652	-0.1372	0.1123	0.0004	0.0114	-1.4396	30.6506	0.8094***
France	1652	-0.1395	0.0861	0.0006	0.0127	-1.1266	15.6205	0.8959***
Germany	1652	-0.1391	0.1097	0.0006	0.0130	-0.8212	13.9849	0.9075***
Italy	1652	-0.1983	0.0828	0.0007	0.0147	-2.2375	28.6184	0.8746***
Japan	1652	-0.0727	0.0712	0.0003	0.0113	-0.1682	5.2951	0.9420***
UK	1652	-0.1290	0.1013	0.0003	0.0122	-1.1507	19.5720	0.8495***
USA	1652	-0.1277	0.0897	0.0006	0.0106	-1.1539	26.3138	0.8198***

Note

[***] signify 1% significance level.

Asymmetries in return distributions are confirmed by these findings, offering a strong incentive to use the BK-18 approach–relative to the DY-12 time-invariant approach–to examine the dynamic connection between emerging and developed markets stocks. Over the studied period, the BRIC composite index and all countries except for Brazil and Russia recorded positive mean returns.

## 5. Empirical results

We report and discuss the main findings of the study in this section. Within the time-frequency space, we first present intriguing findings on the overall spillover connectedness of the composite and constituent indices of BRIC and the G7 equities markets. Second, we examine the spillover connectedness within market blocs. Finally, we highlight the pairwise connectedness of these markets to reveal significant implications for portfolio management.

### 5.1. Time- and frequency-domain analysis

The time-frequency spectrum analysis helps to assess the presence or otherwise of contagion by accounting for the evolvement of total connectedness across time. We advance our analysis by examining the spillover effects between the composite and constituent indices of BRIC and G7 equities across frequencies. This decomposition accounts for market participants’ diverse expectations and desires across investment horizons. The short-, medium- and intermediate-term spillovers, classified into five frequency bands (intraweek, week-fortnight, fortnight-month, month-quarter, and quarter and beyond), in the markets under study are reported in Tables 2–4, in order of BRIC and G7 markets, BRIC and its constituents only, and G7 only.

**Table 2:** Overall and net spillover indices across frequency bands for BRIC and G7 equities.

	BRIC	Brazil	Russia	India	China	Canada	France	Germany	Italy	Japan	UK	USA	FROM_ABS	FROM_WTH
**Band: 3.14~0.79; Approximately 1~4 days**														
BRIC	17.48	8.33	2.98	9.08	5.23	4.53	4.09	3.66	3.04	1.68	4.47	3.48	4.21	5.83
Brazil	10.99	22.66	2.62	4.30	1.03	7.19	4.97	4.11	4.45	0.95	5.12	5.84	4.30	5.94
Russia	5.83	4.15	30.73	2.10	0.86	2.83	3.03	2.95	2.25	0.88	3.05	1.81	2.48	3.43
India	12.16	4.13	1.45	25.74	1.52	3.95	3.74	3.53	2.73	0.99	4.30	3.11	3.47	4.79
China	11.56	1.83	0.94	2.47	44.68	1.24	1.01	0.85	0.69	2.42	1.05	1.24	2.11	2.92
Canada	5.06	6.03	1.53	3.60	0.51	19.62	7.25	6.37	6.26	0.81	8.51	11.46	4.78	6.61
France	3.79	3.21	1.46	2.77	0.37	5.79	15.98	14.14	11.22	0.49	10.30	5.44	***4*.*91***	***6*.*80***
Germany	3.62	2.86	1.56	2.80	0.31	5.50	14.88	16.94	10.84	0.41	10.00	5.38	4.85	6.70
Italy	3.41	3.36	1.27	2.38	0.26	5.71	13.24	12.22	18.95	0.46	10.40	5.02	4.81	6.65
Japan	3.61	2.21	1.18	2.08	1.98	4.25	3.62	3.75	3.06	35.93	3.68	5.10	2.88	3.98
UK	4.38	3.40	1.49	3.32	0.41	6.91	10.67	9.73	9.14	0.86	16.50	5.25	4.63	6.40
USA	4.46	5.48	1.01	3.28	0.63	13.85	7.91	7.34	6.71	0.73	7.39	22.68	4.90	6.77
TO_ABS	5.74	3.75	1.46	3.18	1.09	5.15	6.20	5.72	5.03	0.89	5.69	4.43	**48.33**	
TO_WTH	7.93	5.19	2.02	4.40	1.51	7.12	8.57	7.91	6.96	1.23	7.87	6.12		**66.83**
Net	1.525	-0.547	-1.020	-0.285	-1.018	0.363	1.285	0.873	0.222	-1.988	1.061	-0.470		
**Band: 0.79~0.39; Approximately 4~8 days**														
BRIC	3.46	1.96	0.81	1.52	0.76	1.25	1.04	0.99	0.76	0.35	1.09	1.02	0.96	7.38
Brazil	1.96	3.89	0.62	0.51	0.15	1.20	0.80	0.69	0.65	0.19	0.74	0.81	0.69	5.31
Russia	2.28	1.79	6.14	0.72	0.29	1.26	1.22	1.14	0.87	0.36	1.22	0.86	***1*.*00***	***7*.*66***
India	2.70	1.19	0.47	4.50	0.24	1.19	0.99	0.96	0.68	0.25	1.15	1.00	0.90	6.90
China	2.68	0.60	0.32	0.59	7.27	0.44	0.37	0.33	0.25	0.51	0.37	0.46	0.58	4.41
Canada	0.71	0.88	0.39	0.30	0.05	2.65	1.12	1.03	0.83	0.20	1.17	1.60	0.69	5.28
France	0.59	0.63	0.33	0.29	0.04	1.11	2.36	2.04	1.68	0.15	1.64	1.07	0.80	6.11
Germany	0.55	0.55	0.34	0.28	0.04	1.02	2.19	2.48	1.67	0.13	1.55	1.06	0.78	6.00
Italy	0.45	0.55	0.29	0.23	0.02	0.93	1.86	1.71	2.59	0.13	1.51	0.89	0.71	5.46
Japan	1.11	0.74	0.47	0.50	0.32	1.11	0.94	0.89	0.74	4.77	1.09	1.21	0.76	5.82
UK	0.69	0.71	0.41	0.36	0.05	1.46	1.92	1.75	1.59	0.26	2.79	1.22	0.87	6.65
USA	0.48	0.61	0.25	0.19	0.03	1.36	0.96	0.91	0.65	0.16	0.85	2.42	0.54	4.11
TO_ABS	1.18	0.85	0.39	0.46	0.16	1.03	1.12	1.04	0.86	0.22	1.03	0.93	**9.29**	
TO_WTH	9.06	6.52	3.00	3.51	1.26	7.86	8.55	7.95	6.62	1.71	7.90	7.14		**71.09**
Net	0.221	0.159	-0.608	-0.444	-0.412	0.337	0.318	0.255	0.151	-0.536	0.163	0.396		
**Band: 0.39~0.10; Approximately 8~32 days**														
BRIC	2.97	1.71	0.73	1.25	0.62	1.09	0.92	0.88	0.67	0.31	0.95	0.90	0.84	7.45
Brazil	1.68	3.32	0.55	0.42	0.12	1.03	0.68	0.60	0.55	0.16	0.63	0.69	0.59	5.27
Russia	2.07	1.65	5.40	0.63	0.25	1.17	1.12	1.06	0.80	0.33	1.12	0.80	***0*.*92***	***8*.*15***
India	2.34	1.06	0.44	3.75	0.20	1.05	0.88	0.86	0.60	0.22	1.01	0.89	0.80	7.09
China	2.32	0.54	0.30	0.50	6.05	0.40	0.34	0.30	0.23	0.45	0.34	0.41	0.51	4.53
Canada	0.61	0.76	0.35	0.24	0.04	2.22	0.96	0.89	0.70	0.17	0.99	1.35	0.59	5.23
France	0.50	0.55	0.29	0.22	0.03	0.95	1.98	1.72	1.41	0.13	1.38	0.91	0.67	6.01
Germany	0.47	0.48	0.31	0.22	0.03	0.87	1.83	2.08	1.40	0.12	1.30	0.90	0.66	5.88
Italy	0.38	0.47	0.26	0.18	0.01	0.79	1.56	1.43	2.15	0.11	1.26	0.75	0.60	5.35
Japan	0.99	0.67	0.43	0.42	0.26	0.97	0.82	0.78	0.65	4.02	0.96	1.05	0.67	5.94
UK	0.59	0.62	0.37	0.28	0.04	1.26	1.65	1.51	1.36	0.23	2.36	1.05	0.75	6.65
USA	0.40	0.52	0.22	0.15	0.02	1.13	0.81	0.77	0.55	0.14	0.72	2.00	0.45	4.03
TO_ABS	1.03	0.75	0.35	0.38	0.14	0.89	0.96	0.90	0.74	0.20	0.89	0.81	**8.04**	
TO_WTH	9.17	6.70	3.14	3.35	1.21	7.94	8.59	8.00	6.61	1.76	7.91	7.20		**71.57**
Net	0.194	0.160	-0.563	-0.420	-0.373	0.304	0.290	0.238	0.142	-0.469	0.141	0.356		
**Band: 0.10~0.05; Approximately 32~64 days**														
BRIC	0.60	0.35	0.15	0.25	0.12	0.22	0.19	0.18	0.14	0.06	0.19	0.18	0.17	7.46
Brazil	0.34	0.67	0.11	0.08	0.02	0.21	0.14	0.12	0.11	0.03	0.13	0.14	0.12	5.27
Russia	0.42	0.34	1.09	0.13	0.05	0.24	0.23	0.22	0.16	0.07	0.23	0.16	***0*.*19***	***8*.*26***
India	0.47	0.22	0.09	0.75	0.04	0.21	0.18	0.17	0.12	0.05	0.20	0.18	0.16	7.13
China	0.47	0.11	0.06	0.10	1.21	0.08	0.07	0.06	0.05	0.09	0.07	0.08	0.10	4.56
Canada	0.12	0.15	0.07	0.05	0.01	0.44	0.19	0.18	0.14	0.03	0.20	0.27	0.12	5.22
France	0.10	0.11	0.06	0.04	0.01	0.19	0.40	0.34	0.28	0.03	0.28	0.18	0.14	5.98
Germany	0.09	0.10	0.06	0.04	0.01	0.17	0.37	0.42	0.28	0.02	0.26	0.18	0.13	5.86
Italy	0.08	0.10	0.05	0.04	0.00	0.16	0.31	0.29	0.43	0.02	0.25	0.15	0.12	5.32
Japan	0.20	0.14	0.09	0.08	0.05	0.20	0.17	0.16	0.13	0.81	0.19	0.21	0.13	5.96
UK	0.12	0.13	0.07	0.06	0.01	0.25	0.33	0.30	0.27	0.05	0.47	0.21	0.15	6.65
USA	0.08	0.10	0.05	0.03	0.00	0.23	0.16	0.15	0.11	0.03	0.15	0.40	0.09	4.01
TO_ABS	0.21	0.15	0.07	0.07	0.03	0.18	0.19	0.18	0.15	0.04	0.18	0.16	**1.62**	
TO_WTH	9.20	6.74	3.17	3.31	1.20	7.96	8.60	8.02	6.61	1.77	7.91	7.21		**71.69**
Net	0.039	0.033	-0.115	-0.086	-0.076	0.062	0.059	0.049	0.029	-0.095	0.029	0.072		
**Band: 0.05~0.00; Approximately 64~Infinite days**														
BRIC	0.30	0.17	0.07	0.12	0.06	0.11	0.09	0.09	0.07	0.03	0.10	0.09	0.08	7.46
Brazil	0.17	0.33	0.06	0.04	0.01	0.10	0.07	0.06	0.06	0.02	0.06	0.07	0.06	5.26
Russia	0.21	0.17	0.55	0.06	0.02	0.12	0.12	0.11	0.08	0.03	0.11	0.08	***0*.*09***	***8*.*27***
India	0.24	0.11	0.04	0.38	0.02	0.11	0.09	0.09	0.06	0.02	0.10	0.09	0.08	7.13
China	0.23	0.05	0.03	0.05	0.60	0.04	0.03	0.03	0.02	0.05	0.03	0.04	0.05	4.56
Canada	0.06	0.08	0.04	0.02	0.00	0.22	0.10	0.09	0.07	0.02	0.10	0.14	0.06	5.22
France	0.05	0.06	0.03	0.02	0.00	0.10	0.20	0.17	0.14	0.01	0.14	0.09	0.07	5.98
Germany	0.05	0.05	0.03	0.02	0.00	0.09	0.18	0.21	0.14	0.01	0.13	0.09	0.07	5.86
Italy	0.04	0.05	0.03	0.02	0.00	0.08	0.16	0.14	0.21	0.01	0.13	0.08	0.06	5.32
Japan	0.10	0.07	0.04	0.04	0.03	0.10	0.08	0.08	0.07	0.40	0.10	0.11	0.07	5.97
UK	0.06	0.06	0.04	0.03	0.00	0.13	0.17	0.15	0.14	0.02	0.24	0.11	0.08	6.65
USA	0.04	0.05	0.02	0.01	0.00	0.11	0.08	0.08	0.05	0.01	0.07	0.20	0.05	4.01
TO_ABS	0.10	0.08	0.04	0.04	0.01	0.09	0.10	0.09	0.07	0.02	0.09	0.08	**0.81**	
TO_WTH	9.20	6.74	3.17	3.31	1.20	7.96	8.60	8.02	6.61	1.77	7.91	7.21		**71.70**
Net	0.020	0.017	-0.058	-0.043	-0.038	0.031	0.030	0.024	0.015	-0.047	0.014	0.036		

**Table 3:** Spillover indices across frequency bands for BRIC and its constituents.

	BRIC	Brazil	Russia	India	China	FROM_ABS	FROM_WTH
**Band: 3.14~0.79; Approximately 1~4 days**							
BRIC	28.67	12.85	4.86	15.06	8.78	***8*.*31***	***11*.*88***
Brazil	18.93	41.13	4.65	7.28	1.70	6.51	9.31
Russia	8.60	6.05	45.50	3.15	1.28	3.82	5.46
India	19.09	5.89	2.29	39.91	2.53	5.96	8.52
China	13.57	1.88	1.10	2.99	52.01	3.91	5.59
TO_ABS	12.04	5.33	2.58	5.69	2.86	**28.50**	
TO_WTH	17.21	7.63	3.69	8.14	4.08		**40.75**
Net	3.728	-1.175	-1.238	-0.264	-1.051		
**Band: 0.79~0.39; Approximately 4~8 days**							
BRIC	5.72	3.18	1.29	2.56	1.28	***1*.*66***	***11*.*75***
Brazil	3.35	6.95	1.11	0.86	0.24	1.11	7.87
Russia	3.34	2.57	8.99	1.09	0.43	1.49	10.51
India	4.27	1.85	0.71	7.04	0.41	1.45	10.23
China	3.20	0.70	0.36	0.74	8.48	1.00	7.07
TO_ABS	2.83	1.66	0.70	1.05	0.47	**6.71**	
TO_WTH	20.02	11.74	4.91	7.43	3.33		**47.43**
Net	1.170	0.547	-0.791	-0.397	-0.529		
**Band: 0.39~0.10; Approximately 8~32 days**							
BRIC	4.94	2.81	1.15	2.14	1.05	***1*.*43***	***11*.*72***
Brazil	2.86	5.89	0.98	0.69	0.20	0.94	7.74
Russia	3.03	2.36	7.84	0.95	0.38	1.34	11.00
India	3.73	1.66	0.65	5.92	0.34	1.28	10.45
China	2.80	0.65	0.33	0.64	7.08	0.88	7.24
TO_ABS	2.48	1.50	0.62	0.89	0.39	**5.88**	
TO_WTH	20.33	12.25	5.09	7.26	3.22		**48.15**
Net	1.052	0.551	-0.722	-0.390	-0.491		
**Band: 0.10~0.05; Approximately 32~64 days**							
BRIC	1.00	0.57	0.23	0.43	0.21	***0*.*29***	***11*.*71***
Brazil	0.57	1.18	0.20	0.14	0.04	0.19	7.71
Russia	0.62	0.48	1.58	0.19	0.08	0.27	11.11
India	0.75	0.34	0.13	1.19	0.07	0.26	10.50
China	0.57	0.13	0.07	0.13	1.42	0.18	7.28
TO_ABS	0.50	0.30	0.13	0.18	0.08	**1.19**	
TO_WTH	20.40	12.36	5.13	7.21	3.19		**48.31**
Net	0.214	0.115	-0.147	-0.081	-0.101		
**Band: 0.05~0.00; Approximately 64~Infinite days**							
BRIC	0.50	0.28	0.12	0.21	0.11	***0*.*14***	***11*.*71***
Brazil	0.29	0.59	0.10	0.07	0.02	0.09	7.70
Russia	0.31	0.24	0.79	0.10	0.04	0.14	11.12
India	0.38	0.17	0.07	0.59	0.03	0.13	10.50
China	0.28	0.07	0.03	0.06	0.71	0.09	7.28
TO_ABS	0.25	0.15	0.06	0.09	0.04	**0.60**	
TO_WTH	20.41	12.37	5.14	7.21	3.19		**48.32**
Net	0.107	0.057	-0.074	-0.041	-0.050		

**Table 4:** Spillover indices across frequency bands for G7 equities.

	Canada	France	Germany	Italy	Japan	UK	USA	FROM_ABS	FROM_WTH
**Band: 3.14~0.79; Approximately 1~4 days**									
Canada	24.83	9.37	8.25	8.09	1.09	10.94	14.70	7.49	9.88
France	6.92	18.84	16.69	13.27	0.59	12.19	6.47	***8*.*02***	***10*.*58***
Germany	6.53	17.43	19.80	12.73	0.49	11.77	6.35	7.90	10.43
Italy	6.71	15.36	14.19	21.90	0.57	12.13	5.89	7.84	10.34
Japan	5.49	4.69	4.81	3.93	42.99	4.76	6.42	4.30	5.67
UK	8.42	12.96	11.84	11.15	1.05	19.91	6.41	7.40	9.77
USA	16.97	9.76	9.06	8.29	0.95	9.14	27.42	7.74	10.21
TO_ABS	7.29	9.94	9.26	8.21	0.68	8.71	6.61	**50.69**	
TO_WTH	9.62	13.11	12.22	10.83	0.89	11.49	8.72		**66.88**
Net	-0.201	1.920	1.363	0.371	-3.624	1.301	-1.132		
**Band: 0.79~0.39; Approximately 4~8 days**									
Canada	3.34	1.42	1.31	1.05	0.25	1.48	2.05	1.08	9.32
France	1.34	2.79	2.42	1.99	0.17	1.95	1.31	1.31	11.33
Germany	1.23	2.56	2.90	1.97	0.15	1.83	1.28	1.29	11.13
Italy	1.10	2.16	1.98	2.99	0.14	1.75	1.06	1.17	10.11
Japan	1.32	1.10	1.05	0.88	5.73	1.28	1.45	1.01	8.74
UK	1.81	2.33	2.14	1.94	0.30	3.37	1.52	***1*.*43***	***12*.*39***
USA	1.66	1.16	1.10	0.79	0.20	1.04	2.93	0.85	7.33
TO_ABS	1.21	1.53	1.43	1.23	0.17	1.33	1.24	**8.15**	
TO_WTH	10.43	13.23	12.34	10.64	1.49	11.51	10.70		**70.34**
Net	0.129	0.220	0.140	0.062	-0.840	-0.102	0.390		
**Band: 0.39~0.10; Approximately 8~32 days**									
Canada	2.77	1.19	1.11	0.88	0.21	1.24	1.72	0.91	9.32
France	1.14	2.33	2.02	1.66	0.15	1.63	1.12	1.10	11.35
Germany	1.03	2.13	2.42	1.64	0.13	1.53	1.09	1.08	11.11
Italy	0.92	1.79	1.65	2.46	0.12	1.46	0.90	0.98	10.04
Japan	1.13	0.94	0.90	0.75	4.77	1.10	1.24	0.87	8.92
UK	1.55	1.98	1.82	1.64	0.26	2.84	1.32	***1*.*22***	***12*.*60***
USA	1.36	0.96	0.91	0.65	0.17	0.87	2.41	0.70	7.24
TO_ABS	1.02	1.29	1.20	1.03	0.15	1.12	1.05	**6.86**	
TO_WTH	10.49	13.24	12.37	10.61	1.53	11.50	10.85		**70.59**
Net	0.113	0.183	0.122	0.056	-0.718	-0.106	0.350		
**Band: 0.10~0.05; Approximately 32~64 days**									
Canada	0.55	0.24	0.22	0.18	0.04	0.25	0.34	0.18	9.32
France	0.23	0.47	0.40	0.33	0.03	0.33	0.22	0.22	11.36
Germany	0.21	0.43	0.48	0.33	0.03	0.31	0.22	0.22	11.11
Italy	0.18	0.36	0.33	0.49	0.02	0.29	0.18	0.20	10.02
Japan	0.23	0.19	0.18	0.15	0.95	0.22	0.25	0.17	8.96
UK	0.31	0.40	0.37	0.33	0.05	0.57	0.27	***0*.*25***	***12*.*64***
USA	0.27	0.19	0.18	0.13	0.03	0.17	0.48	0.14	7.22
TO_ABS	0.20	0.26	0.24	0.21	0.03	0.22	0.21	**1.37**	
TO_WTH	10.50	13.24	12.38	10.61	1.54	11.50	10.88		**70.65**
Net	0.023	0.037	0.025	0.011	-0.144	-0.022	0.071		
**Band: 0.05~0.00; Approximately 64~Infinite days**									
Canada	0.28	0.12	0.11	0.09	0.02	0.12	0.17	0.09	9.32
France	0.11	0.23	0.20	0.17	0.02	0.16	0.11	0.11	11.36
Germany	0.10	0.21	0.24	0.16	0.01	0.15	0.11	0.11	11.11
Italy	0.09	0.18	0.16	0.25	0.01	0.15	0.09	0.10	10.02
Japan	0.11	0.10	0.09	0.08	0.48	0.11	0.12	0.09	8.97
UK	0.16	0.20	0.18	0.16	0.03	0.29	0.13	***0*.*12***	***12*.*65***
USA	0.14	0.10	0.09	0.07	0.02	0.09	0.24	0.07	7.22
TO_ABS	0.10	0.13	0.12	0.10	0.02	0.11	0.11	**0.69**	
TO_WTH	10.50	13.24	12.38	10.61	1.54	11.50	10.88		**70.65**
Net	0.011	0.018	0.012	0.006	-0.072	-0.011	0.036		

Note that from the spillover tables, ‘TO_ABS’ measures return spillovers from market/country *j* to other markets. ‘FROM_ABS’ measures return spillovers from other markets to market *j*. ‘TO_WTH’ measures return spillovers from market *j* to other markets, including from own innovations to country *k*. ‘FROM_WTH’ measures return spillovers from other markets to market *j*, including from own innovations to market *k* (Owusu Junior, Alagidede et al., [14]; Tiwari et al., [31,57]). The largest market contributions per frequency band are in bold italics. A positive (negative) ‘Net’ suggests that the country/market is a net transmitter (recipient) of shocks.

#### 5.1.1. Overall spillover connectedness

From the overall spillover indices for all the studied markets (see Table 2), we find that the magnitude of spillovers reduces with increasing frequencies. Thus, in the short-term (intraweek), spillovers are higher than in the medium- to long-term horizons. For instance, the return spillover within the first band, 3.14~0.79, which approximates 1~4 days, is 48.33%. This spillover reduces to 9.29%, 8.04%, 1.62%, and 0.81% respectively through the second to fifth bands (0.79~0.00) frequency bands. A similar observation is made for the spillovers among both BRIC and G7 markets when studied in solitude, suggesting that both emerging and developed markets respond quickly to shocks in the first few trading days. Our findings are consistent with those of Mensi et al. [60], who use a similar methodology and find that short-term spillovers are more significant than intermediate-term spillovers for Islamic and BRICS economies.

It is worthy of notice that individual markets are also influenced by their own shocks. For instance, the Russian, Chinese, and Japanese stock markets receive over 30% of spillovers from their own markets and are predominant at high frequencies. This means that internal shocks also form a significant consideration in asset allocation decisions, corroborating the findings of Khalfaoui, Boutahar, and Boubaker [61] and Mensi et al. [60].

When the BRIC and G7 markets are studied together, the composite BRIC index proves to be the greatest transmitter of shocks to all markets. The constituent markets, however, hardly present themselves as great shock contributors. These findings are suggestive of the relative strength of integrated markets as opposed to individual economies. Our findings corroborate Owusu Junior, Adam et al.’s [5] observation that the contribution of the BRIC constituents to the BRIC index is significantly positive; thus, together, it is not surprising that the BRIC index transmits the highest shocks to both emerging and developed markets across all frequencies.

From the individual markets, we find France, Germany, and the UK as the largest contributors of shocks in the high-frequency bands (3.14~0.39), notably in the short term. In the mid-term, Canada and the US join the group of largest spillover contributors, with the UK leaving the group. In the long term (low-frequency band), France, Canada, Germany, and the US form the group of largest shock transmitters. On the other hand, across all spillover bands, we find Japan and China to be the smallest contributors to the shocks between the studied markets. This implies that from the BRIC (G7) economies, the Chinese (Japanese) market has fewer shocks to present to developed (emerging) markets.

From Table 2, we reveal the net transmitters and recipients of spillovers among BRIC and G7 markets. The results suggest that in the short term (3.14~0.79), the net transmitters (recipients) of spillovers across the BRIC and G7 markets are the BRIC index, Canada, France, Germany, and the UK (Brazil, Russia, India, and China). Aside from India, all other constituents of BRIC remained net spillover recipients. All G7 markets except Japan were found to be net transmitters of shocks in the mid-to-long-term periods. The findings suggest that the nature of spillovers across and within the BRIC and G7 markets is time-frequency-dependent, which is consistent with the HMH [23–25] the AMH [22], and the CMH [34]. This observation accentuates the conclusion of Mensi et al. [60] who revealed that volatility spillovers among Islamic and BRICS equities were time-frequency-dependent.

In terms of recipients of market volatilities, we report that France endures the highest amount of spillover from the studied markets in the short term (3.14~0.79). Between bands 2 and 5, Russia is found to be the recipient of the largest spillover among the BRIC and G7 markets. The Chinese stock market proved consistently to be the lowest shock recipient; it is not surprising since it also emerged as the market with fewer shocks to present although it is highly affected by its own shocks.

**Fig 2:**
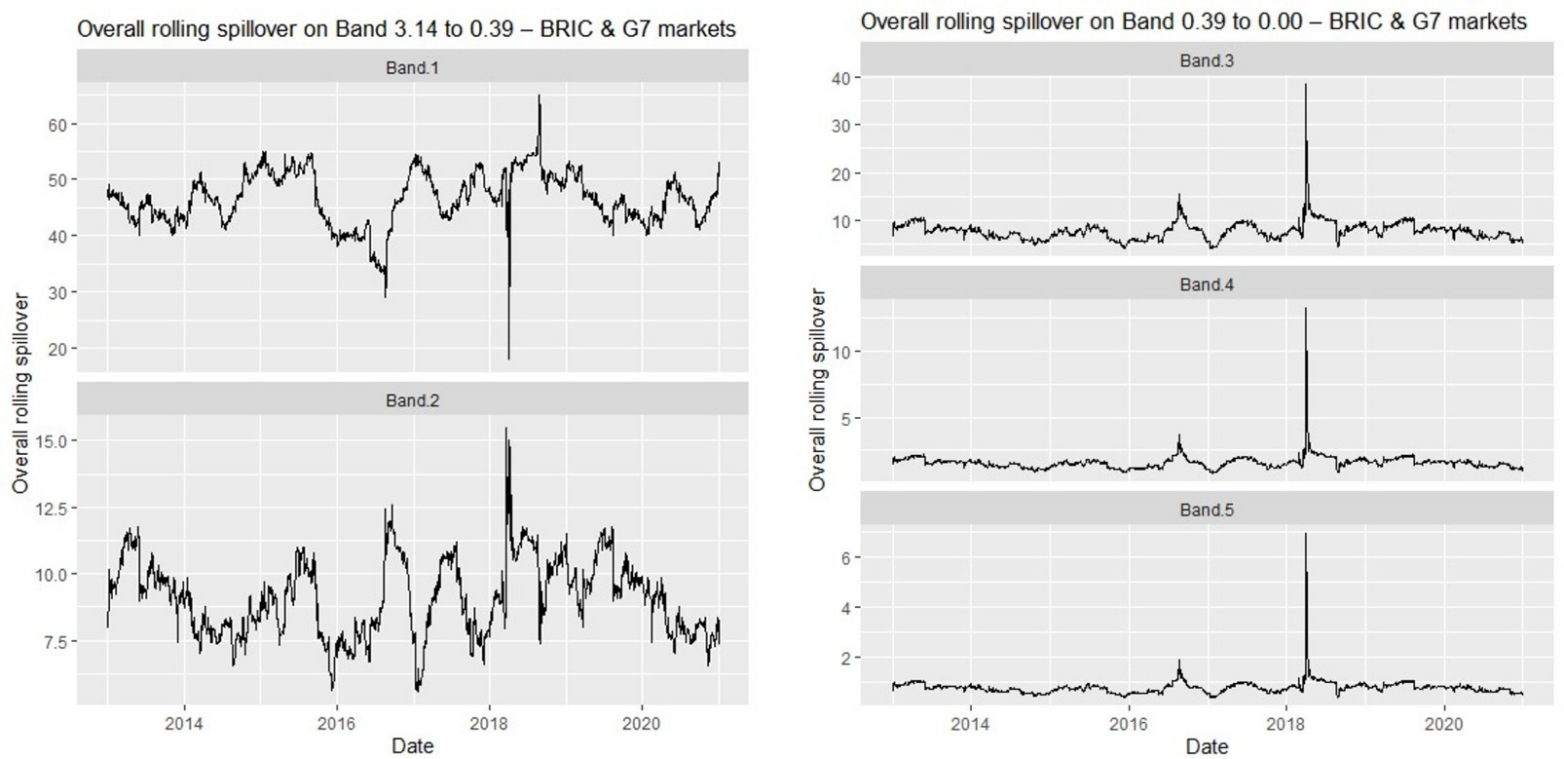
Overall rolling spillovers between BRIC and G7 equities.

To substantiate our findings, we present the time-frequency subtleties of the return volatility among the BRIC and G7 stocks in Fig 2.

We notice intense volatility spillovers in the short term. The spillovers across the frequency bands come in differing magnitudes. We notice variations in the spillover but largely between 40% and 50% in the short-term (band 1), with a sharp drop to less than 30% and 20% respectively in 2017 and 2019. These calendar dates could be respectively traced to the persistent impact of Brexit and the US-China trade tension. Again, in 2019, an upsurge above 65% was revealed, and this corroborates the era of the US-China trade tension. Surprisingly, over the studied period, we find no significant change in the average spillover between the BRIC and G7 markets in the COVID-19 pandemic era.

In spillover band 2, volatility clusters are prevalent but largely between 5.0% and 12.5%, with an upsurge above 15% in 2019, which was more intense (over 35%) in the spillover band 0.39~0.50 (intermediate-term). Through the mid-term and the long term, consistent upsurges in spillovers–with decreasing magnitudes–were found in 2017 and 2019, but the upsurge in 2019 was more intense. This is not surprising since the countries in question, China and the US, for the significant event in the 2019 calendar year (US-China trade tension) are studied together and belong to separate economic classes (i.e., developed and emerging respectively).

Thus, we infer contagion across all spillover bands (Fig 2) on two occasions. Our results reveal that spillovers in 2017 and 2019 are attributable to contagion, where we identify substantial increases in spillover connectedness between BRIC and G7 markets. This corroborates Forbes and Rigobon’s [62,63] definition of contagion. For example, in 2019, at the high-frequency band (in the short-term), we observe surging volatilities (about 65%) between the markets and reduces to about 16% in the frequency 0.79~0.39, increases to about 38% in band 3, and finally reduce through bands 4 to 5. Following Mensi et al. [60], we attribute this contagion to the slowdown in economic activities experienced by China in 2017 and/or the substantial losses borne by global investors on 24/06/2016 due to Brexit [64]. We find the sources of the inferred contagion to be France and the UK (Canada and the US) in the short term (medium-to-long term).

#### 5.1.2. Connectedness within market blocs

Next, we isolate the two broad markets to study the transmission of volatilities within them. The spillovers are reported in Tables 3 and 4 for the BRIC and its constituents, and the G7 equities respectively. Note that the general observations–vis-à-vis the intensity of spillovers across frequency bands–made from the study of the market blocs together hold for the separate markets.

From the BRIC and its constituent equities only, we find that the BRIC index offers the greatest spillovers in the very short-term (i.e., within band 3.14~0.79), and is followed by Russia and Brazil. In frequency bands 2–5, Brazil consistently follows the BRIC index as the greatest contributor of spillovers to the individual BRIC equities. This observation confirms the findings of Owusu Junior, Adam et al. [5], who employ the wavelet methodology and reveal that in terms of market shocks, when the BRIC index and its constituent indices are studied together, the BRIC index is the first responder to shocks, followed by Brazil. It is not surprising that across all frequencies, we find the BRIC and Brazilian equities to be net transmitters of shocks while all other constituents remain net shock recipients. Except at the frequency band 3.14~0.79, where China and Russia emerged as the greatest (least) shock recipients (transmitters), the Chinese market remained consistently the greatest (least) shock recipient (transmitter) to BRIC markets across the remaining frequencies.

Now, we turn to the G7 markets only (see Table 4) to compare developed and emerging markets. This aids in assessing diversification prospects across economic blocs.

We find France and Germany as the greatest spillover transmitters across all frequency bands, with Japan being the lowest shock transmitter. France receives the largest spillover in the short term, frequency band 3.14~0.79. In frequency bands, 2–5, the UK (US) emerges as the recipient of the largest (smallest) shocks.

**Fig 3:**
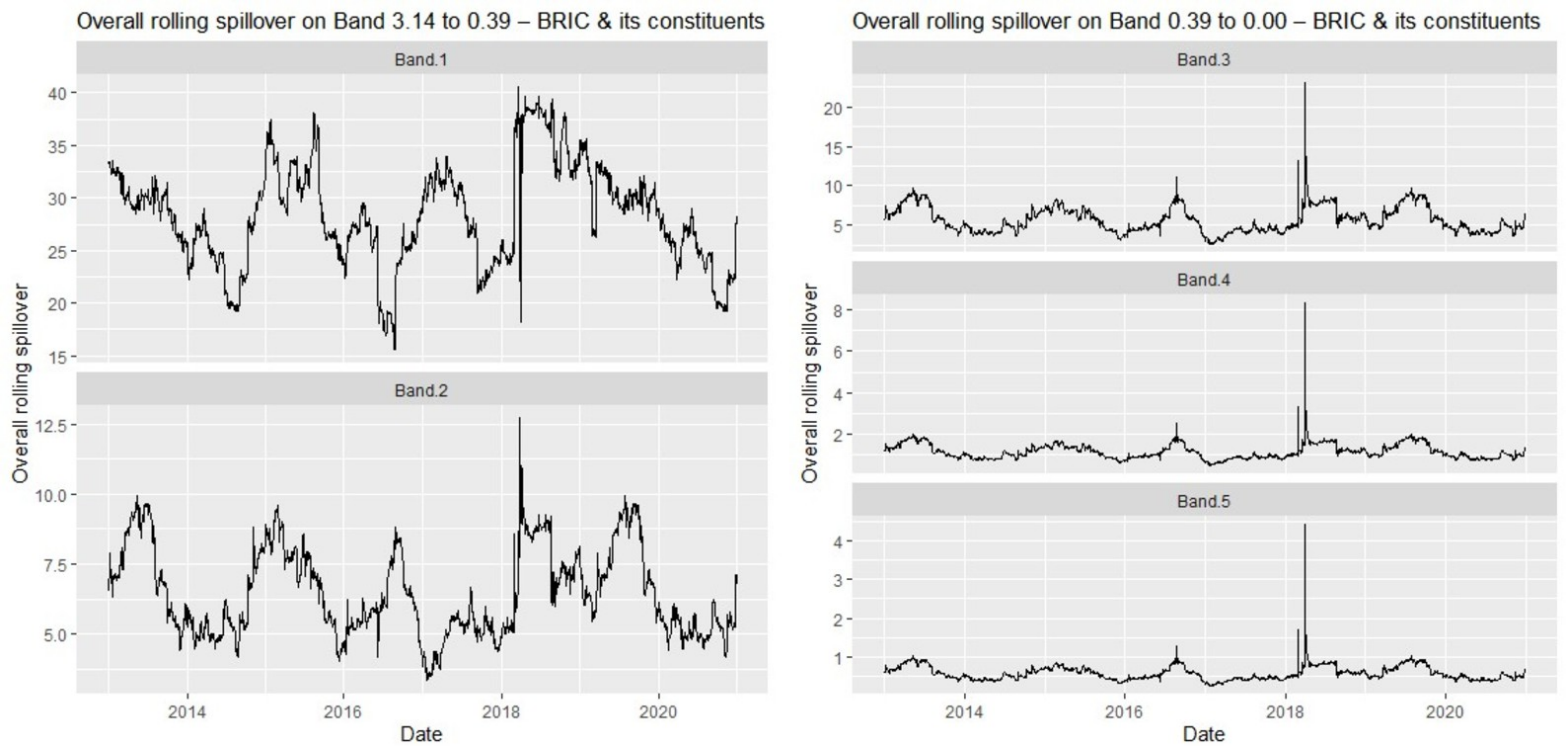
Overall rolling spillovers between BRIC composite index and its constituents.

**Fig 4:**
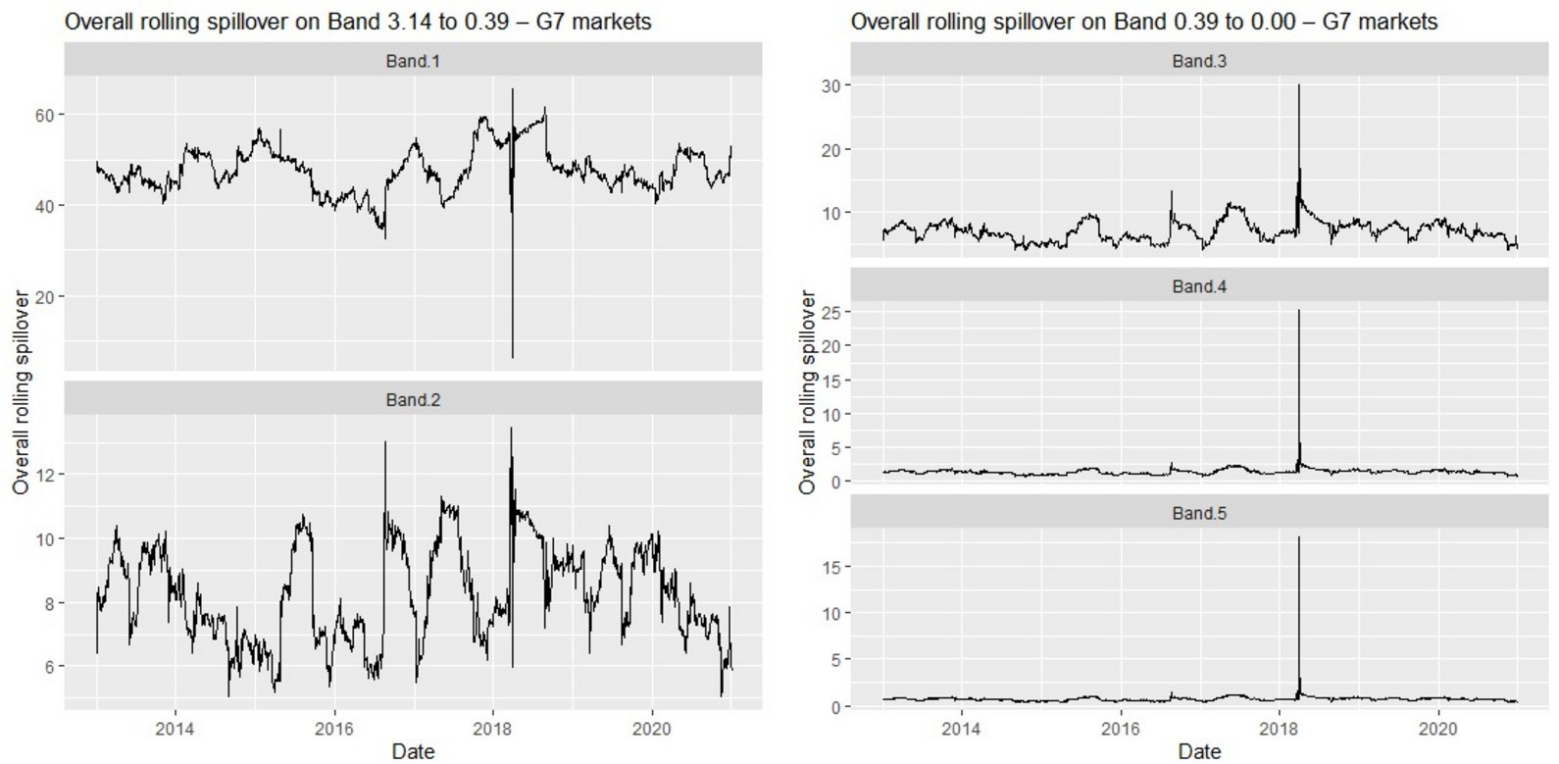
Overall rolling spillovers between G7 equities.

We confirm our findings for each market bloc with the rolling window spillover plots in Fig 3 (BRIC) and Fig 4 (G7).

The rolling spillovers in the frequency domain (see Figs 3 and 4) substantiate the initial results when the BRIC and G7 markets were studied together. For example, volatility clusters are still observable in each market bloc’s rolling spillover plots, and the intensified spillovers across the medium-and-long-term for all markets also persist for each market bloc. However, note that the magnitude of spillovers for BRIC markets only is lower than when studied together with the G7, suggesting that BRIC stock markets may be less volatile than those of the G7. We reiterate that the returns offered by assets from emerging markets may be more consistent relative to those from advanced markets [4]. This is confirmed since the magnitudes of spillovers for G7 stocks only are higher than those of BRIC. Therefore, this signals possible diversification prospects between BRIC and G7 markets.

#### 5.1.3. Pairwise connectedness

We report pairwise connectedness to examine diversification prospects between pairs of BRIC and G7 equities. The pairs of the BRIC index and its constituents are reported in Table 5 to examine the connectedness of each constituent to the development of the BRIC composite index, which assesses the degree of market integration between the constituents. The pairs of BRIC and G7 equities are summarised in Table 6. We provide pairwise plots in S1 and S2 Figs.

**Table 5:** Frequency-domain pairs of BRIC composite index and its constituents.

Band: 3.14~0.79; Approximately 1~4 days				Total BRIC spillover contributors
BRIC-Brazil	BRIC-Russia	BRIC-India	BRIC-China	All constituents
-0.22142	-0.2374	-0.25745	-0.52767	
**Band: 0.79~0.39; Approximately 4~8 days**				
BRIC-Brazil	BRIC-Russia	BRIC-India	BRIC-China	All constituents
-0.00012	-0.12219	-0.09844	-0.15964	
**Band: 0.39~0.10; Approximately 8~32 days**				
BRIC-Brazil	BRIC-Russia	BRIC-India	BRIC-China	Excluding Brazil
0.002434	-0.11157	-0.09093	-0.14164	
**Band: 0.10~0.05; Approximately 32~64 days**				
BRIC-Brazil	BRIC-Russia	BRIC-India	BRIC-China	Excluding Brazil
6.13E-04	-2.28E-02	-1.86E-02	-2.87E-02	
**Band: 0.05~0.00; Approximately 64~Infinite days**				
BRIC-Brazil	BRIC-Russia	BRIC-India	BRIC-China	Excluding Brazil
3.10E-04	-1.14E-02	-9.33E-03	-1.44E-02	

Note: A positive net shows a net transmitter position for the first variable.

A negative net shows a net recipient position for the second variable.

**Table 6:** Frequency-domain pairs of BRIC and G7 equities.

**Band: 3.14~0.79; Approximately 1~4 days**						
Brazil-Canada	Brazil-France	Brazil-Germany	Brazil-Italy	Brazil-Japan	Brazil-UK	Brazil-USA
0.096239	0.146465	0.103967	0.090717	-0.10535	0.143718	0.030134
Russia-Canada	Russia-France	Russia-Germany	Russia-Italy	Russia-Japan	Russia-UK	Russia-USA
0.108505	0.130621	0.115269	0.081607	-0.02481	0.130404	0.066657
India-Canada	India-France	India-Germany	India-Italy	India-Japan	India-UK	India-USA
0.029031	0.080713	0.060453	0.029685	-0.09092	0.081228	-0.01439
China-Canada	China-France	China-Germany	China-Italy	China-Japan	China-UK	China-USA
0.061206	0.05306	0.0452	0.036081	0.036125	0.053655	0.051319
**Band: 0.79~0.39; Approximately 4~8 days**						
Brazil-Canada	Brazil-France	Brazil-Germany	Brazil-Italy	Brazil-Japan	Brazil-UK	Brazil-USA
0.026362	0.0136	0.011624	0.007676	-0.04611	0.003005	0.017028
Russia-Canada	Russia-France	Russia-Germany	Russia-Italy	Russia-Japan	Russia-UK	Russia-USA
0.072224	0.074199	0.06602	0.048089	-0.00931	0.067971	0.050854
India-Canada	India-France	India-Germany	India-Italy	India-Japan	India-UK	India-USA
0.073757	0.058245	0.056499	0.037889	-0.02087	0.065623	0.067566
China-Canada	China-France	China-Germany	China-Italy	China-Japan	China-UK	China-USA
0.032523	0.027383	0.024295	0.019464	0.015968	0.026953	0.03572
**and: 0.39~0.10; Approximately 8~32 days**						
Brazil-Canada	Brazil-France	Brazil-Germany	Brazil-Italy	Brazil-Japan	Brazil-UK	Brazil-USA
0.022538	0.011292	0.010193	0.006371	-0.04222	0.000726	0.014057
Russia-Canada	Russia-France	Russia-Germany	Russia-Italy	Russia-Japan	Russia-UK	Russia-USA
0.068084	0.069472	0.06256	0.045103	-0.00859	0.062646	0.048192
India-Canada	India-France	India-Germany	India-Italy	India-Japan	India-UK	India-USA
0.067837	0.054213	0.052923	0.035108	-0.01608	0.060622	0.061788
China-Canada	China-France	China-Germany	China-Italy	China-Japan	China-UK	China-USA
0.029448	0.025162	0.022447	0.01772	0.015395	0.024585	0.032362
**Band: 0.10~0.05; Approximately 32~64 days**						
Brazil-Canada	Brazil-France	Brazil-Germany	Brazil-Italy	Brazil-Japan	Brazil-UK	Brazil-USA
4.53E-03	2.25E-03	2.06E-03	1.27E-03	-8.63E-03	5.42E-05	2.80E-03
Russia-Canada	Russia-France	Russia-Germany	Russia-Italy	Russia-Japan	Russia-UK	Russia-USA
1.40E-02	1.43E-02	1.29E-02	9.27E-03	-1.76E-03	1.28E-02	9.92E-03
India-Canada	India-France	India-Germany	India-Italy	India-Japan	India-UK	India-USA
1.39E-02	1.11E-02	1.09E-02	7.19E-03	-3.15E-03	1.24E-02	1.26E-02
China-Canada	China-France	China-Germany	China-Italy	China-Japan	China-UK	China-USA
6.00E-03	5.15E-03	4.60E-03	3.62E-03	3.18E-03	5.02E-03	6.60E-03
**Band: 0.05~0.00; Approximately 64~Infinite days**						
Brazil-Canada	Brazil-France	Brazil-Germany	Brazil-Italy	Brazil-Japan	Brazil-UK	Brazil-USA
2.26E-03	1.13E-03	1.03E-03	6.35E-04	-4.32E-03	2.43E-05	1.40E-03
Russia-Canada	Russia-France	Russia-Germany	Russia-Italy	Russia-Japan	Russia-UK	Russia-USA
7.01E-03	7.14E-03	6.45E-03	4.64E-03	-8.81E-04	6.42E-03	4.97E-03
India-Canada	India-France	India-Germany	India-Italy	India-Japan	India-UK	India-USA
6.95E-03	5.57E-03	5.44E-03	3.60E-03	-1.57E-03	6.21E-03	6.32E-03
China-Canada	China-France	China-Germany	China-Italy	China-Japan	China-UK	China-USA
3.01E-03	2.58E-03	2.30E-03	1.81E-03	1.59E-03	2.51E-03	3.30E-03

Note: Numbers are in percentage.

The pairwise results between BRIC composite index and its constituents suggest that in the early trading days, up to a week (1~8 days), all constituents make a significant contribution to the development of the BRIC index. Thus, the BRIC countries are integrated towards the BRIC index across all frequencies, with Brazil being the only exception in the mid-to-long-term periods. Intuitively, the composite spillovers are significantly contributed by the individual stock markets, stressing the linkages between the BRIC composite index and its members. Our findings support the conclusions of Owusu Junior, Adam et al. [5], who advocate high coherences between the BRIC index and its constituent indices across all investment horizons. This may hinder (promote) diversification (hedge) opportunities between BRIC markets and their composite index.

From the pairs of BRIC and G7 equities, we find that, generally, across all time scales, BRIC markets are net-pairwise transmitters of spillovers with G7 markets save Japan. It is important to note that the net-pairwise spillovers reduce in magnitude with increasing frequencies. In line with Jena, Tiwari, Dash, and Aikins Abakah [65], the findings (positive net-pairwise spillovers) are suggestive of the fact that diversification between BRIC and G7 equities can be profitable relative to between BRIC markets themselves. In the short term, we find India as the only BRIC country to be a recipient of net-pairwise spillover from the US. Notwithstanding, the resilience of the BRIC economies to provide diversification benefits for developed markets cannot go unnoticed.

Summarily, it is worthily noting that diverse causes or channels of the effect of the intensified spillovers during Brexit and the US-China trade tension could be identified. For Brexit, the exit of Britain from the European Union might have caused a negative signal to international investors concerning their portfolio holdings. In responding to such a negative signal, all responses in the short term are attributable to transitory factors, which are likely to affect cross-market correlations [65,66], which then causes a significant change in the fundamental connectedness between developed and developing markets. Owusu Junior, Frimpong et al. [34], Bossman [23], and Bossman [24] note that market dynamics and connectedness in the medium- (long-) term are attributable to key events (fundamental factors). In the medium term, key events in the market also drive the connectedness between assets and such connectedness could last throughout the event. We spot such connectedness in the era of Brexit and the US-China trade tension. In the long-term, where markets are saturated with information flow within and across markets, spillovers and connectedness are attributable to the fundamental or longstanding relationships between markets (assets and/or asset classes). Information flows and spillovers are predominant in crises periods. Resultantly, due to the action(s) of rational, albeit irrational investors, any policy uncertainties that hit the market would be reacted to, leading to the emergence of short-lived connections within and across financial markets [65,66].

As regards the COVID-19 era, where no significant change in cross-market connectedness was spotted, we advance that the current strategy adopted by emerging economies–towards economic growth and development–may be a contributory factor. As noted by Fokuo and Ochieng [67], the recent ties between African economies and top emerging economies are critical to the growth of the world economy at large despite the recent tensions in the global economy occasioned by either the US-China trade disputes or the actions from other members–such as Russia–from the BRIC market bloc. Notable developing economies from Africa and top-developing markets like China, Russia, and the UAE are members of these new alliances. These alliances are critical owing to the growth prospects possessed by these economies. Suffice to say, despite the tensions between top-developed (like the G7) and top-emerging economies (like the BRIC) in recent periods, it may be unsurprising that when notable crises hit the global economy, the overall effect may be less than expected. However, the reason for the less connectivity of both regions in the COVID-19 pandemic period is partly attributed to their diverse market structures, price formation, price discovery, transaction and timing cost, information and disclosure etc., elicited by markets participants heightened during the COVID-19 to inhibit market integration. Consequently, the COVID-19 pandemic had an asymmetric rather than analogous impact on most financial markets as divulged by prior studies [5,11,23,24,34] which precipitate into markets segregation. Hence, to some extent, the preserved interdependence between the BRIC and G7 markets in the COVID-19 era may be understood from this direction.

### 5.2. Robustness

**Fig 5:**
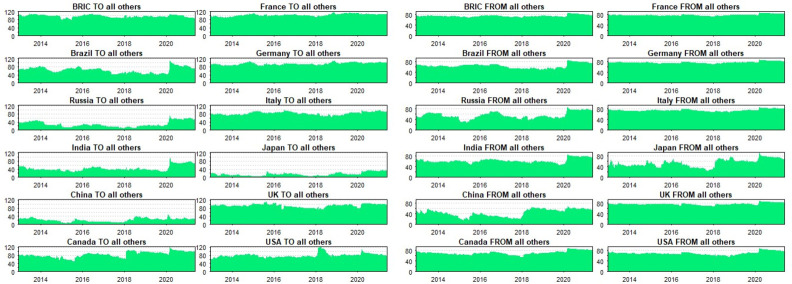
Directional spillovers between BRIC and G7stock markets. (a) and (b) are in respect of ‘TO’ and ‘FROM’ spillovers in the system.

**Fig 6:**
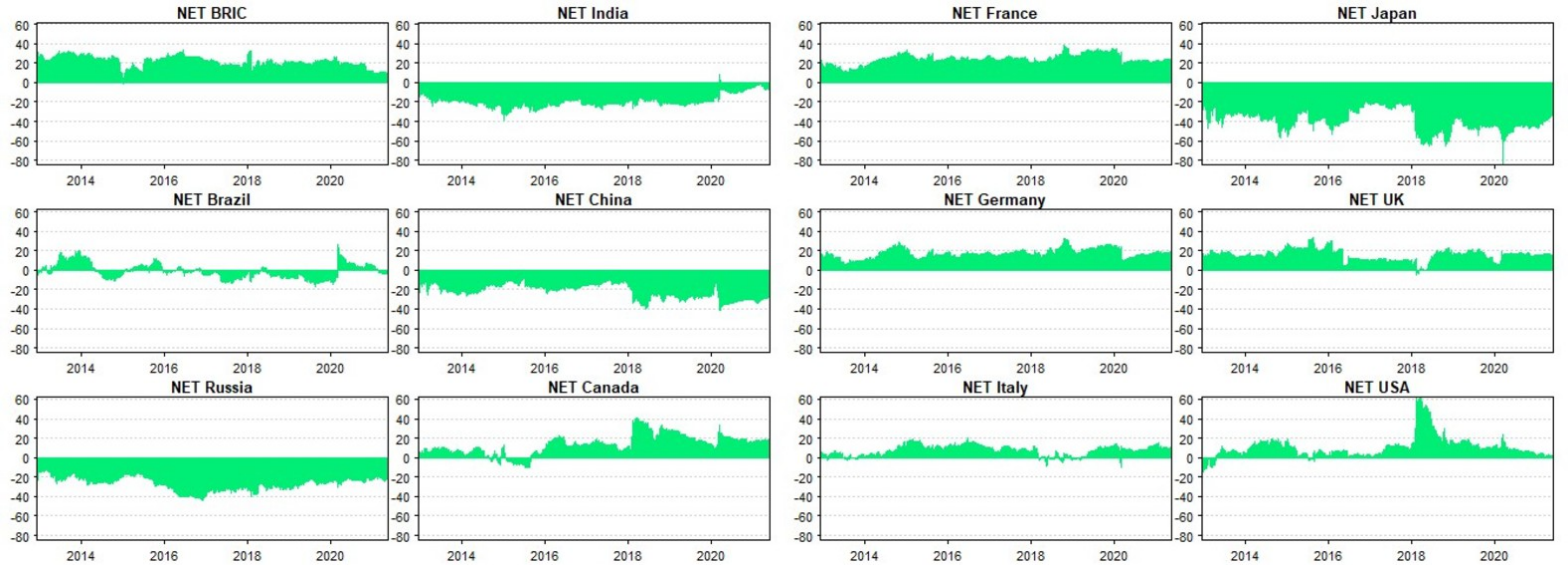
Net directional spillovers between BRIC and G7 stock markets.

In the spirit of Antonakakis, Chatziantoniou, and Gabauer’s [68] study, we resort to the time-varying parameter vector autoregressive (TVP-VAR) model to assess how robust our results may be to different methods. Antonakakis et al. [68] present comprehensive notes on the TVP-VAR methodology. The TVP-VAR overcomes the problem of randomised specification of rolling-window sizes. In accounting for dynamism, adaptability, and heterogeneity in assets’ connectedness, and examining the frequency-dependent connectedness of BRIC and G7 in our main analysis, we capture rolling window analysis. Considering the relative merit of Antonakakis et al.’s [68] TVP-VAR model, it serves as an appropriate technique to evaluate the robustness of our findings from the Baruník and Křehlík [14] spillover index approach. However, note that the TVP-VAR connectedness approach falls on the static paradigm and hence, does not produce frequency-dependent results; only time-varying estimates are applicable. Consequently, we specifically employ the directional (‘TO’ and ‘FROM’) and net spillover connectedness to assess the robustness of the findings from the BK-18 approach. Fig 5 depicts the directional spillovers between BRIC and G7 markets with their net connectivity revealed by Fig 6.

While we spot a substantial amount of directional spillovers in respect of the BRIC composite index and most of the G7 markets, the directional spillovers are of less magnitude for the constituents of BRIC. This corroborates our previous findings that emerging markets could offer consistent investment returns relative to their counterparts from advanced markets [4]. Moreover, the strength of an integrated market bloc is revealed by the high magnitude of directional spillovers attributable to the BRIC composite index. These findings are further confirmed by Fig 6, which reveals the net directional spillover status of BRIC and G7 markets.

From Fig 6, we confirm and infer the status of various stock markets as diversifiers and safe-havens for others in the system based on market conditions. Indicatively, we find China, India, and Russia as consistent diversifiers (safe-havens) for all G7 economies–save Japan–during tranquil (exuberant) trading periods. Brazil stocks switch positions as net transmitters and recipients of shocks and hence, casts doubts about its consistency in risk management. This finding is not different from the one revealed by the BK-18, where we found that the BRIC index is the first responder to shocks, followed by Brazil. This observation supports the findings of Owusu Junior, Adam et al. [5]. Impliedly, assets from emerging economies could be excellent diversification candidates for those from developed economies. Notwithstanding, Japan’s ability to diversify shocks from all other G7 markets, as revealed by the net spillover (Fig 6) cannot go unnoticed.

With the overwhelming similarities in the results offered by the BK-18 and the TVP-VAR models, we conclude that our findings are robust to new techniques that account for dynamisms in assets’ linkages resulting from the heterogeneous and adaptive behaviour of market participants. At large, the time-varying intricacies between BRIC and G7 markets in recent episodes of financial crises revealed by the BK-18 approach are confirmed by the TVP-VAR connectedness measure.

## 6. Practical implications

### 6.1. Investors and/or market participants

Generally, our results suggest that spillovers largely dominate in the short-term within high frequency/spillover bands 3.14~0.39. That is, the total spillovers existent between BRIC and G7 equities could be attributable to short-term transitory conditions [65,66]. Both markets are more likely to be immune to the shocks presented to financial markets globally during the COVID-19 pandemic, but they are less immune to the impacts of Brexit and the US-China trade tension. These findings are supported by the extant literature such as Heliodoro, Dias, and Alexandre [69], who found no contagion between stocks from Latin America and the US in the COVID-19 era.

Within the intraweek band over the studied period, emerging and developed markets are more responsive to market shocks than in later days. These results are consistent with the EMH such that in the short-term, asset prices fully reflect all pertinent information [70,71], resulting in exuberant market dynamics. Impliedly, investors should be wary of the high co-movement of BRIC and G7 equities in the early trading days (up to a week) in crises periods since diversification, safe-haven, and hedging opportunities may be futile. During turbulent trading days, institutional investors and speculators may have to focus on BRIC and G7 markets in the medium- to long-term horizons, where volatilities are less prevalent.

More importantly, our findings divulge that diversification between BRIC and G7 equities would be viable in the medium- and long-term horizons only. Impliedly, in the intermediate- and long-term horizons, the EMH may not hold as investors might have adapted and rebalanced their portfolios regarding their behavioural intentions. Investors would seek to maximise (mitigate) portfolio returns (risks) across investment horizons [7,23,24] and, hence, should they find themselves in tumult trading periods, it is optimal that they adapt to the heterogenous market responses based on their appetite for risks. This observation is supported by the AMH [22], HMH [23–25,33], and CMH [34].

### 6.2. Regulators and/or governments

We emphasise that the stocks of an integrated emerging-market bloc (such as the BRIC index) possess greater shocks than individual markets in that the constituent markets make significant positive contributions to the aggregate index of the market bloc; thus, integrated emerging market blocs are expected to transmit substantial sum of shocks to developed markets. This explains the high interdependency–between the BRIC index and its constituent indices across all investment horizons–established in the work of Owusu Junior, Adam et al. [5].

One of the stylised facts highlighted by previous results is that there are significant spillovers between the equities markets of the US and those of other nations [2,21], corroborating the fact that “when America sneezes, the whole world catches a cold,” as Adam [53] puts it. With BRIC economies, our findings divulge that middle-income and emerging economies like the BRIC are susceptible to shocks from France and Germany more than the US, and contributes to the reason why the BRIC economies survived despite the capital flight due to tapering by the US Federal Reserve [5]. Therefore, aside from shocks from the US market, portfolio managers and policymakers should pay attention to shocks from other developed economies in addition to those inherently produced by the individual markets from which they hold equities or assets.

## 7. Conclusions

The portfolio risk diversification features of BRIC equity markets are known to be transformed due to the sharp extinction of the concept of the BRIC being synchronised engines of global development, a corollary to the 2007/08 GFC. Recent developments in the global economy (e.g., the 2011/12 EDC, Brexit issues, US-China trade tension, COVID-19 pandemic, etc.), coupled with the crises they come with, warrant both assessments and re-assessments of existing conclusions about market blocs, their extent of integration, portfolio diversification prospects, through appropriate methods that account for the heterogeneous, complex, and adaptable behaviours [26] of market participants. Therefore, the time-frequency spillovers, contagion, and pairwise interrelations between the BRIC index and its members, and between BRIC and G7 economies, were investigated in this study. The Baruník and Křehlík [14] spillover index was used with daily data from 11/12/2012, to 28/05/2021. Overall and net-pairwise spillover connectedness and contagion were assessed together for the BRIC index and its constituents, and a further examination with the G7 equities under a 100-day rolling window and forecast horizon.

Our findings divulged that, together, the BRIC composite index transmits the greatest spillovers to developed markets. Focusing on the individual markets, we found France, Germany, and the UK to be the major providers of shocks in the short term. In the intermediate-term, we explicate that Canada and the US will join the group of countries that contribute the most spillovers to BRIC markets, while the UK will leave the group. France, Canada, Germany, and the United States are the greatest shock broadcasters in the long term. On the other hand, we found that the Chinese (Japanese) market has fewer shocks to present to developed (developing) markets than the BRIC (G7) economies. Our findings vary across time and frequencies, substantiating the heterogeneity, complexity, and adaptability of financial markets, as expounded by the AMH, HMH, and the CMH (see Lo [22]; Bossman [23,24], Owusu Junior, Frimpong, et al. [34]). This emphasised the appropriateness of the econometric approach employed in the study.

Although we found insufficient evidence of significant change in spillovers during the COVID-19 period, the hikes in the connectedness of the BRIC and G7 markets in 2017 and 2019 were found to coincide with the Brexit effect and the US-China trade tension, respectively, making them a contagion rather than interdependence. The net-pairwise spillovers between China and the G7 markets confirmed these contagious spillovers. As Forbes and Rigobon [62,63] explain, contagion exists when a significant change in the connectedness between markets is realised after a shock is experienced by one market. This definition has been employed in notable studies like Bossman et al. [66], Owusu Junior [36], Owusu Junior, Alagidede et al. [58], and Tiwari et al. [31,57]. Our findings advocate that these time- and frequency-dependent contagious spillovers are triggered by France and the UK in the short-term, but in the medium- to long-term, Canada and the US.

Furthermore, the constituents of BRIC contribute significantly to the development of the BRIC composite index, and hence, communicating the extent of integration between the BRIC economies. This finding makes diversification between BRIC equities only unsafe across diverse frequencies, and this substantiates Owusu Junior, Adam, et al.’s [5] conclusion. However, we found diversification opportunities between paired BRIC and G7 equities. Given that all BRIC constituents make a direct contribution to the development of the BRIC, it is important to note that hedge benefits could be ascertained between BRIC equities.

Our findings provide significant implications for effective asset allocation and portfolio management. First, investors and/or market participants with assets covering more than one equities belonging to BRIC need to pay attention to the time- and frequency-connectedness between those equities due to their integration with the BRIC index. Even in portfolios that combine equities from middle-income or emerging economies and developed markets, such as BRIC and G7, investors and fund managers should note that individual markets are also influenced by their own shocks, which must be given attention. That is, international investors should pay critical attention to shocks intrinsic to individual markets when allocating assets.

Second, regulators must note that market regulation strategies should not only focus on factors emanating from a renounced economy like the US only. Given that emerging markets may be more susceptible to shocks from other economies like France and Germany, as revealed by our findings, it would be prudent for regulators to devise comprehensive strategies that take into consideration the overall economic states of other advanced economies rather than focusing on the economic state of the US only.

Finally, emerging markets are noted for their recent strategy of attracting capital flows to build their economies. In line with this, for governments or economies to attract substantial income flows, regulators should pay attention to intrinsic shocks that affect individual markets, and the pairwise and overall connectedness of their assets to those from other markets. Findings from this study should induce policy-makers and governments across the globe to promote effective financial openness for an enhanced international trade flows to boost portfolio diversification.

From the premise laid by this study, future studies could examine conditional spillovers between BRIC markets to reveal how the markets respond to spillovers in conditional tails of the return distribution. The family of quantile regressions and entropy with appropriate decompositions may be relevant.

## Supporting information

S1 Fig Pairs of BRIC composite index and its constituents. (ZIP)

S2 Fig Pairs of BRIC and G7 equities. (ZIP)

S1 Data (ZIP)
