# Supplementary figures and images for: Spillovers and contagion between BRIC and G7 markets: New evidence from time-frequency analysis
Source: PLoS One. 2022 Jul 27;17(7):e0271088. doi: 10.1371/journal.pone.0271088 (PMC9328562; doi:10.1371/journal.pone.0271088)

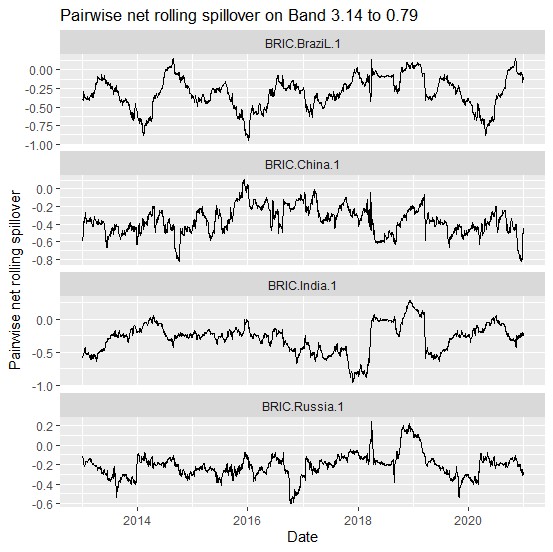

Supplement: S1 Fig — (ZIP) [file pone.0271088.s001.zip › S1_Fig/S1_Fig_a.jpg]

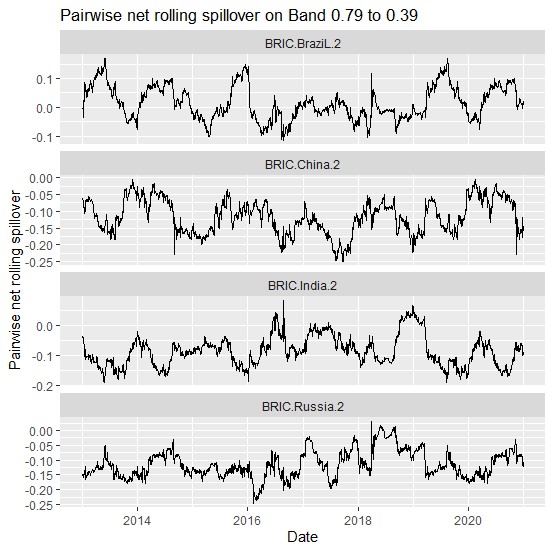

Supplement: S1 Fig — (ZIP) [file pone.0271088.s001.zip › S1_Fig/S1_Fig_b.jpg]

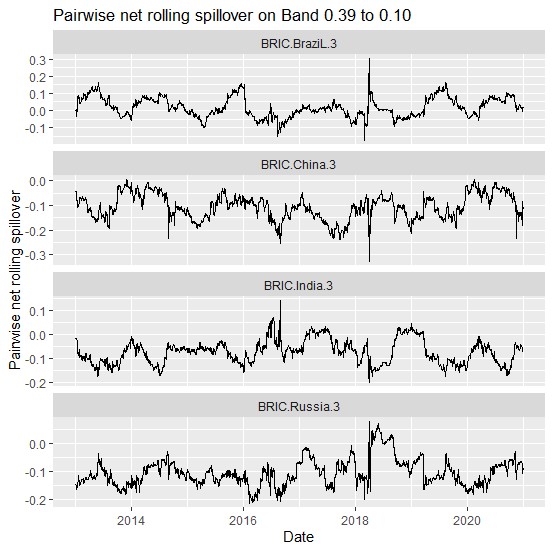

Supplement: S1 Fig — (ZIP) [file pone.0271088.s001.zip › S1_Fig/S1_Fig_c.jpg]

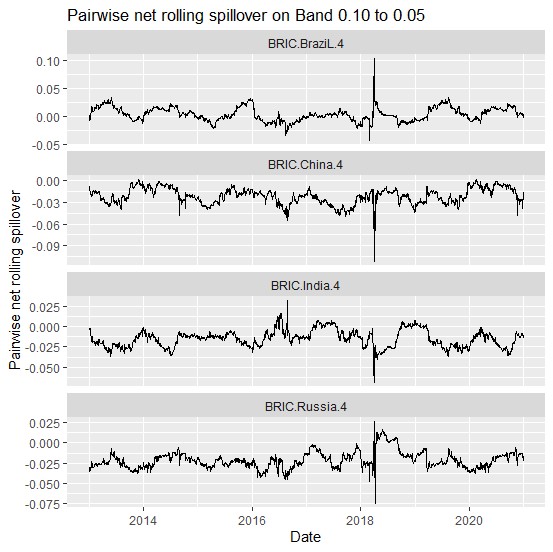

Supplement: S1 Fig — (ZIP) [file pone.0271088.s001.zip › S1_Fig/S1_Fig_d.jpg]

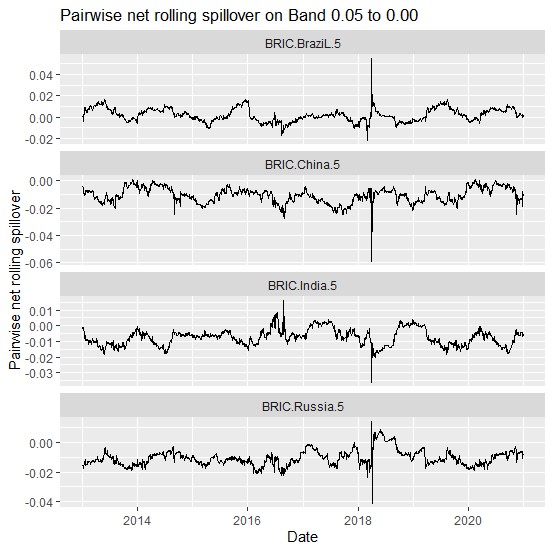

Supplement: S1 Fig — (ZIP) [file pone.0271088.s001.zip › S1_Fig/S1_Fig_e.jpg]

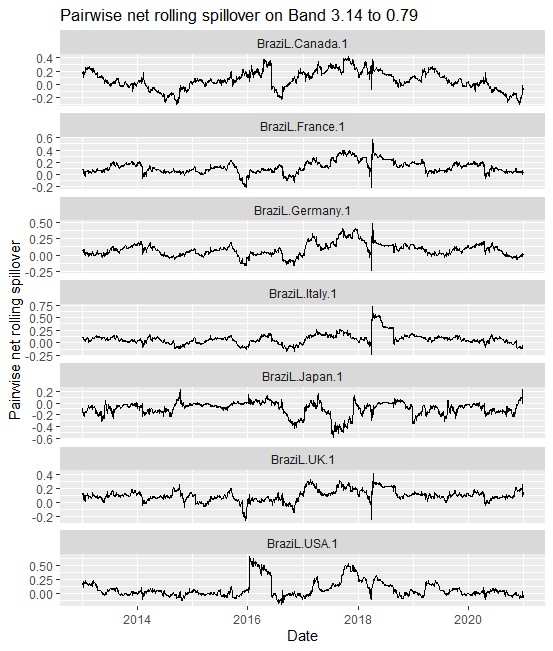

Supplement: S2 Fig — (ZIP) [file pone.0271088.s002.zip › S2_Fig/S2_Fig_a.jpg]

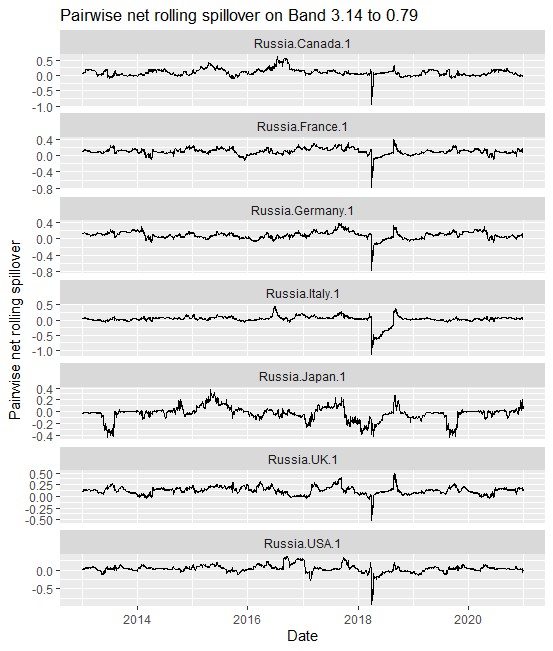

Supplement: S2 Fig — (ZIP) [file pone.0271088.s002.zip › S2_Fig/S2_Fig_b.jpg]

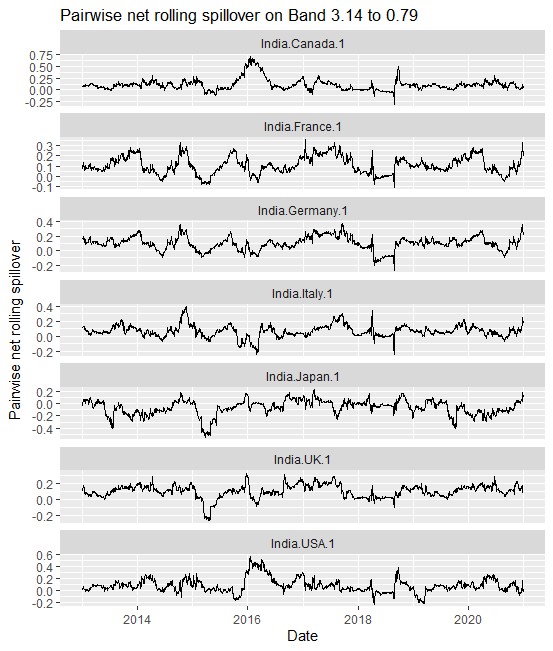

Supplement: S2 Fig — (ZIP) [file pone.0271088.s002.zip › S2_Fig/S2_Fig_c.jpg]

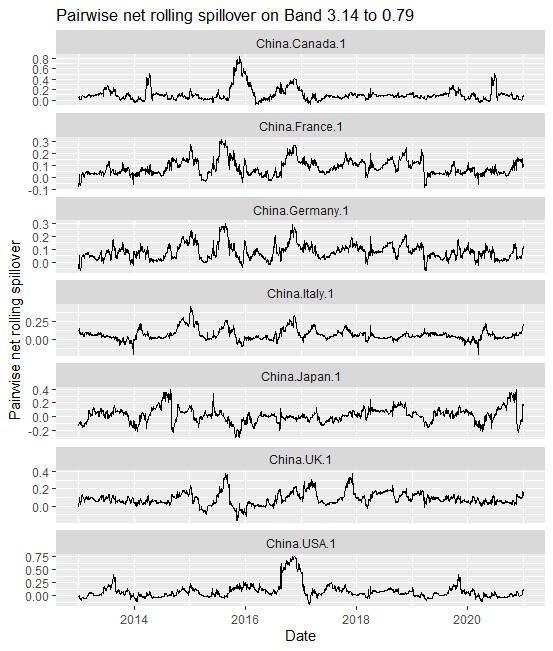

Supplement: S2 Fig — (ZIP) [file pone.0271088.s002.zip › S2_Fig/S2_Fig_d.jpg]

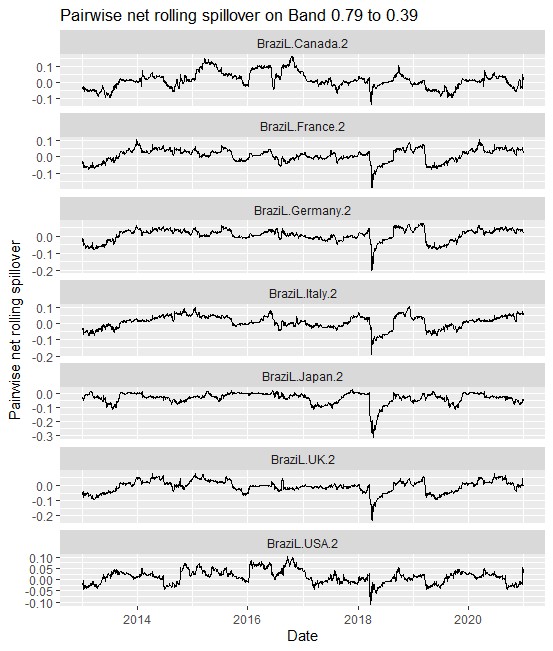

Supplement: S2 Fig — (ZIP) [file pone.0271088.s002.zip › S2_Fig/S2_Fig_e.jpg]

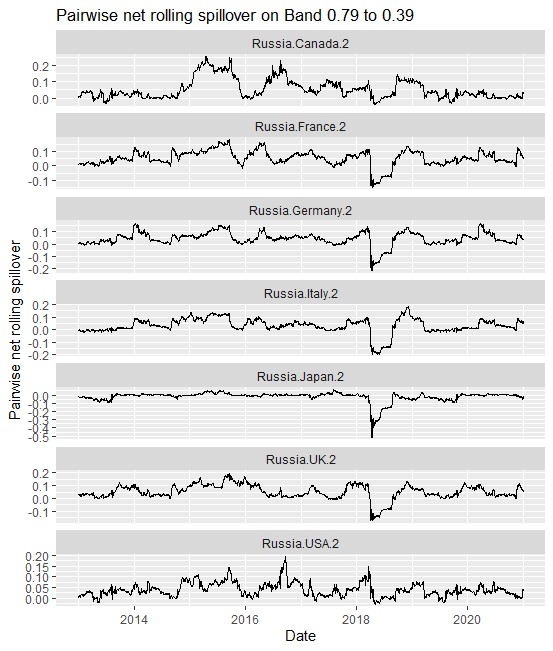

Supplement: S2 Fig — (ZIP) [file pone.0271088.s002.zip › S2_Fig/S2_Fig_f.jpg]

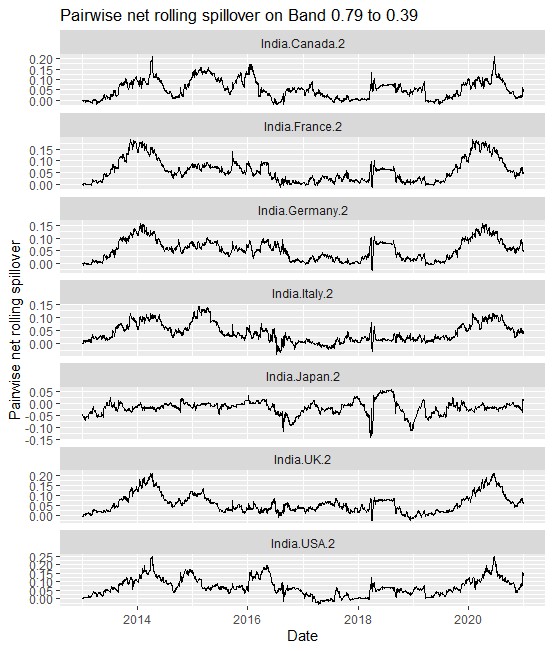

Supplement: S2 Fig — (ZIP) [file pone.0271088.s002.zip › S2_Fig/S2_Fig_g.jpg]

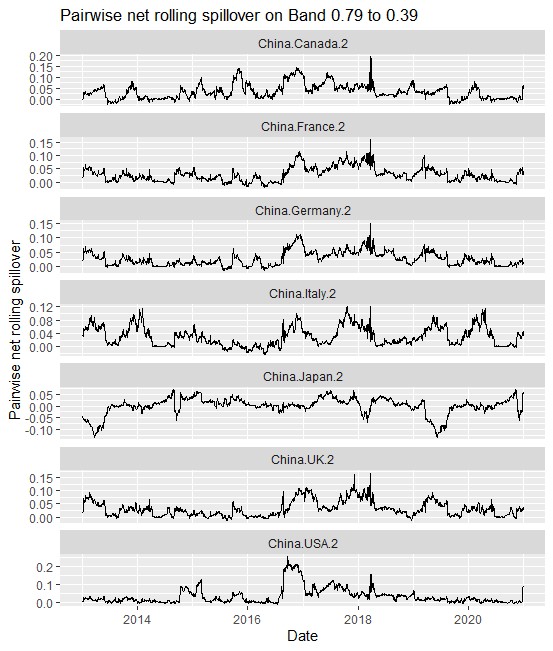

Supplement: S2 Fig — (ZIP) [file pone.0271088.s002.zip › S2_Fig/S2_Fig_h.jpg]

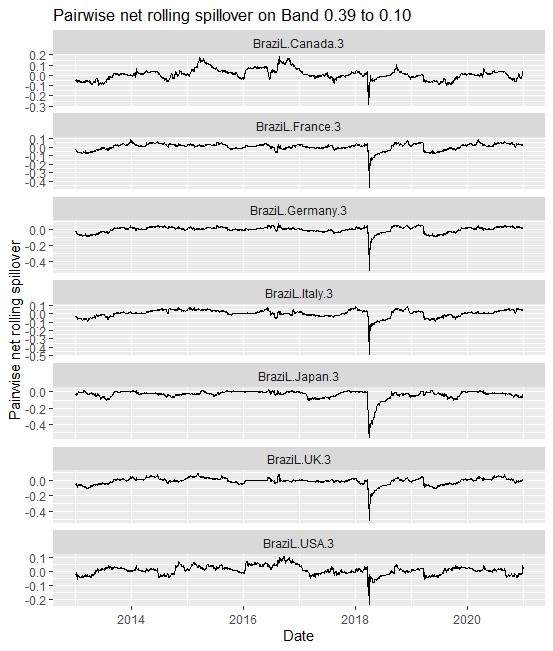

Supplement: S2 Fig — (ZIP) [file pone.0271088.s002.zip › S2_Fig/S2_Fig_i.jpg]

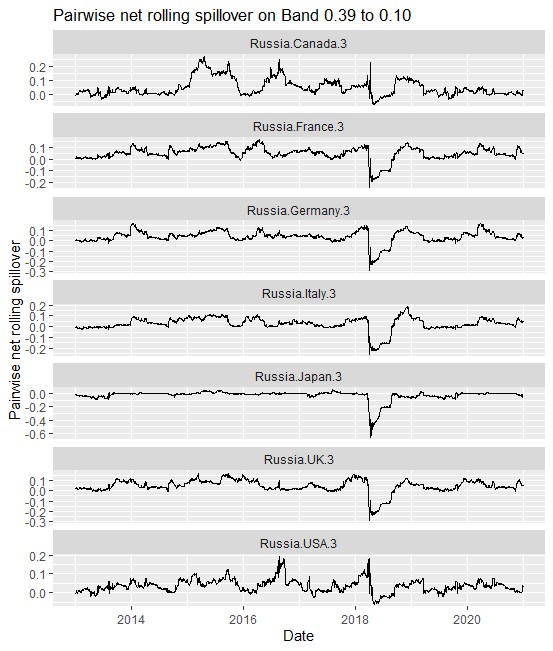

Supplement: S2 Fig — (ZIP) [file pone.0271088.s002.zip › S2_Fig/S2_Fig_j.jpg]

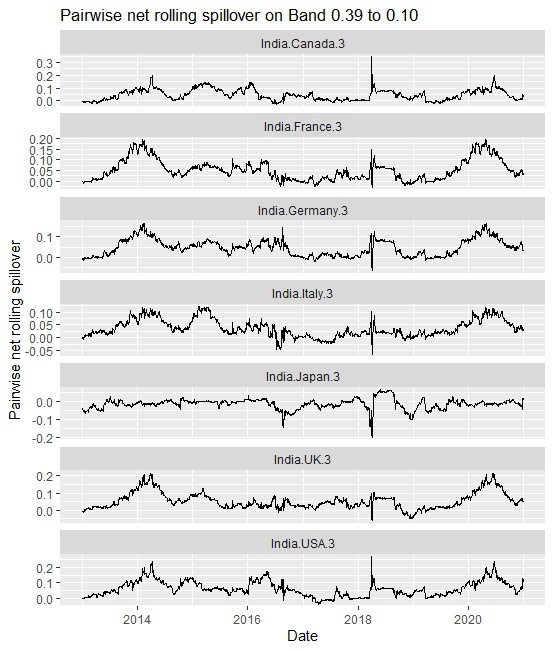

Supplement: S2 Fig — (ZIP) [file pone.0271088.s002.zip › S2_Fig/S2_Fig_k.jpg]

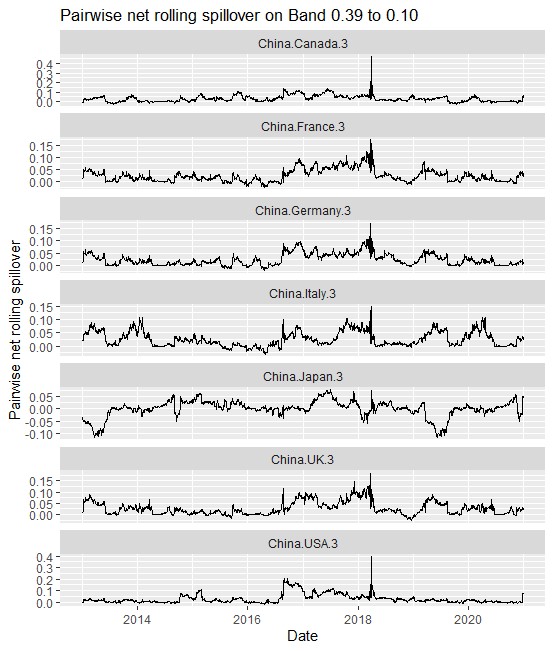

Supplement: S2 Fig — (ZIP) [file pone.0271088.s002.zip › S2_Fig/S2_Fig_l.jpg]

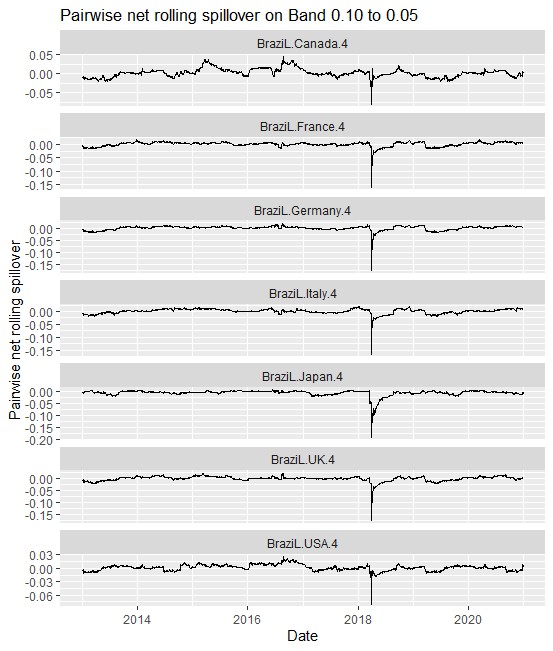

Supplement: S2 Fig — (ZIP) [file pone.0271088.s002.zip › S2_Fig/S2_Fig_m.jpg]

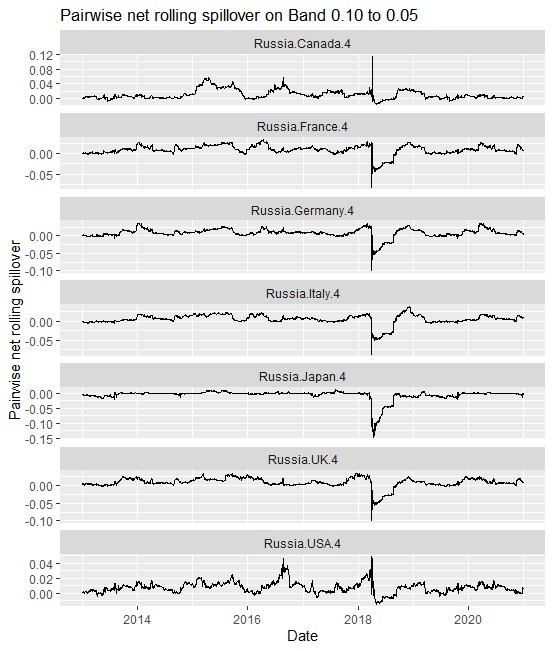

Supplement: S2 Fig — (ZIP) [file pone.0271088.s002.zip › S2_Fig/S2_Fig_n.jpg]

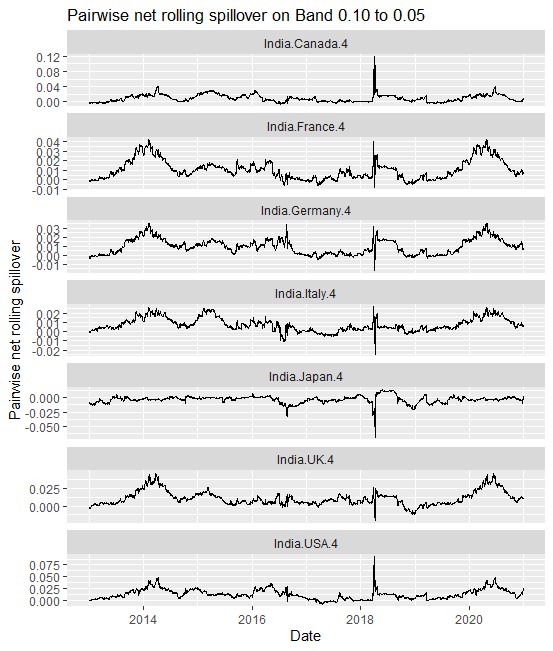

Supplement: S2 Fig — (ZIP) [file pone.0271088.s002.zip › S2_Fig/S2_Fig_o.jpg]

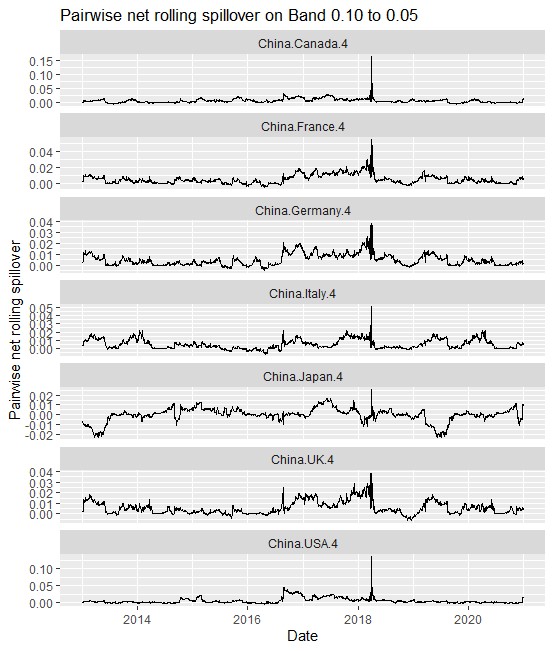

Supplement: S2 Fig — (ZIP) [file pone.0271088.s002.zip › S2_Fig/S2_Fig_p.jpg]

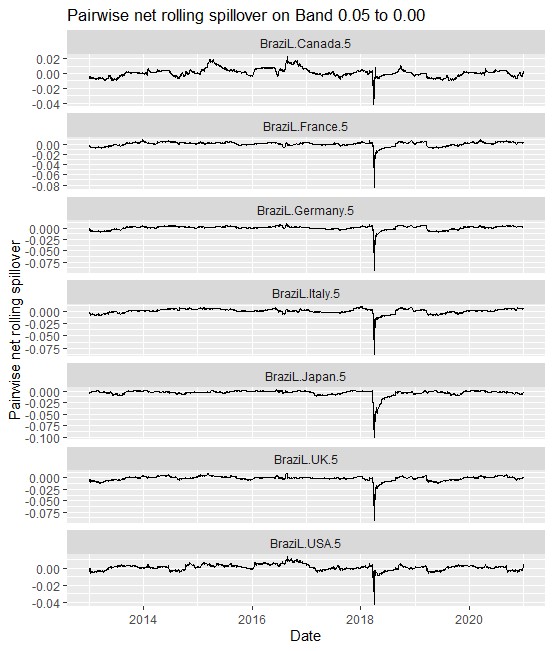

Supplement: S2 Fig — (ZIP) [file pone.0271088.s002.zip › S2_Fig/S2_Fig_q.jpg]

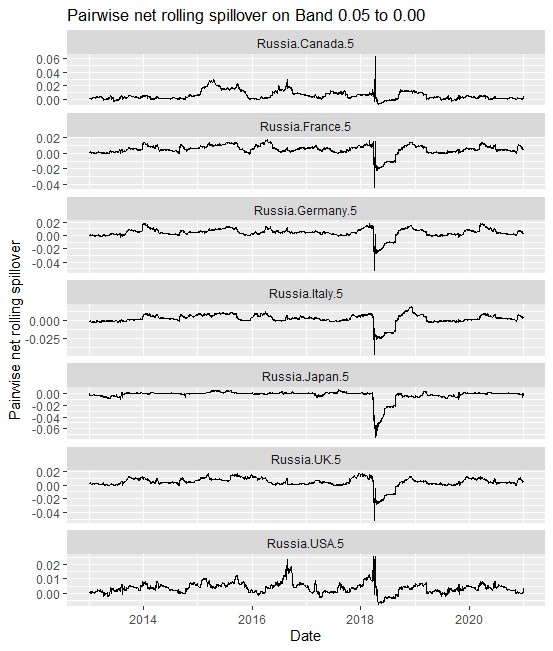

Supplement: S2 Fig — (ZIP) [file pone.0271088.s002.zip › S2_Fig/S2_Fig_r.jpg]

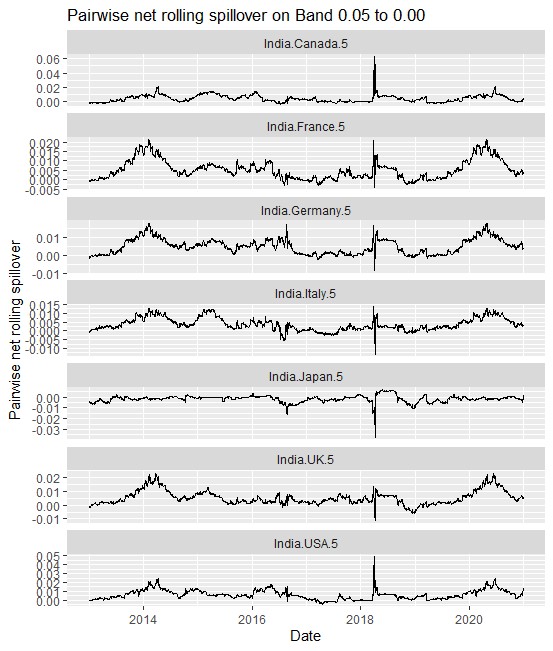

Supplement: S2 Fig — (ZIP) [file pone.0271088.s002.zip › S2_Fig/S2_Fig_s.jpg]

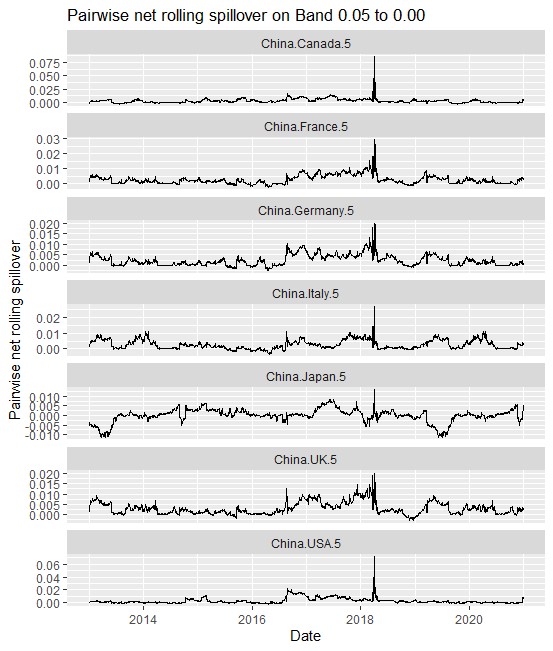

Supplement: S2 Fig — (ZIP) [file pone.0271088.s002.zip › S2_Fig/S2_Fig_t.jpg]
